# Genetic Testing in Egyptian Patients with Inborn Errors of Immunity: a Single-Center Experience

**DOI:** 10.1007/s10875-022-01272-y

**Published:** 2022-04-28

**Authors:** Rabab E. EL Hawary, Safa S. Meshaal, Dalia S. Abd Elaziz, Radwa Alkady, Sohilla Lotfy, Alia Eldash, Aya Erfan, Engy A. Chohayeb, Mai M. Saad, Rania K. Darwish, Jeannette A. Boutros, Nermeen M. Galal, Aisha M. Elmarsafy

**Affiliations:** 1 Clinical Pathology Department, Faculty of Medicine, Cairo University, Cairo, Egypt; 2 Pediatrics Department, Faculty of Medicine, Cairo University, Cairo, Egypt; 3 Chemical Pathology Department, Faculty of Medicine, Cairo University, Cairo, Egypt

**Keywords:** Human inborn errors of immunity, genetic diagnosis, personalized treatment, networking

## Abstract

**Background:**

Inborn errors of immunity (IEI) are a group of heterogeneous disorders with geographic and ethnic diversities. Although IEI are common in Egypt, genetic diagnosis is limited due to financial restrictions. This study aims to characterize the genetic spectrum of IEI patients in Egypt and highlights the adaptation of the molecular diagnostic methods to a resource-limited setting.

**Methods:**

Genetic material from 504 patients was studied, and proper diagnosis was achieved in 282 patients from 246 families. Mutational analysis was done by Sanger sequencing, next-generation sequencing (NGS) targeting customized genes panels, and whole-exome sequencing (WES) according to the patients’ phenotypes and availability of genetic testing.

**Results:**

A total of 194 variants involving 72 different genes were detected with *RAG1/2* genes being the most encountered followed by *DOCK8*, *CYBA*, *LRBA*, *NCF1*, and *JAK3*. Autosomal recessive (AR) inheritance was detected in 233/282 patients (82.6%), X-linked (XL) recessive inheritance in 32/282 patients (11.3%), and autosomal dominant (AD) inheritance in 18/282 patients (6.4%), reflecting the impact of consanguineous marriages on the prevalence of different modes of inheritance and the distribution of the various IEI disorders.

**Conclusion:**

The study showed that a combination of Sanger sequencing in selected patients associated with targeted NGS or WES in other patients is an effective diagnostic strategy for IEI diagnosis in countries with limited diagnostic resources. Molecular testing can be used to validate other nonexpensive laboratory techniques that help to reach definitive diagnosis and help in genetic counseling and taking proper therapeutic decisions including stem cell transplantation or gene therapy.

**Supplementary Information:**

The online version contains supplementary material available at 10.1007/s10875-022-01272-y.

## Introduction

Inborn errors of immunity (IEI) comprise a heterogeneous group of genetic disorders caused by defects in one or more components of the immune system, resulting in a wide spectrum of clinical manifestations and laboratory abnormalities. These patients have an increased susceptibility to infections and higher risks of developing autoimmune diseases and malignancies [1, 2].

A delay in the diagnosis can contribute to the morbidity and mortality, especially the atypical cases where genetic testing becomes an indispensable part of patients’ evaluation. It provides a definitive diagnosis, helps to establish phenotype-genotype correlation, improves decisions of curative interventions, and opens the possibility of genetic counseling for affected families [2, 3].

This study aims to outline the genetic makeup of IEI diseases in Egypt (a country with high rates of consanguinity and limited resources) and describes the achievement in the field of genetic diagnosis of IEI in Egypt in the past decade and the optimum way to use the available genetic tools to reach a refined precise diagnosis.

## Patients and Methods

Among 1496 IEI patients referred between 2010 and 2021 to the Tertiary Primary Immunodeficiency (PID) Referral Center at Cairo University specialized Children’s Hospital (Cairo, Egypt), 1000 patients with specific phenotype/probable diagnosis of IEI according to International Union of Immunological Societies (IUIS) classification [4]/ESID criteria for diagnosis [5] were included for genetic analysis in this study. An informed consent was taken from the patients’ parents or legal guardians and the study was approved by the local institutional review board.

Detailed medical history, clinical evaluation, and laboratory workup were recorded for each patient. Genomic DNA was extracted from the peripheral blood using QIAamp DNA blood Minikit (Qiagen, Germany) according to the manufacturer’s instructions.

Sanger sequencing using earlier established protocols was performed for patients with classical clinical presentations denoting a specific PID phenotype; e.g., severe combined immunodeficiency disorder (SCID) were tested for *RAG1*, *RAG2*, *PNP*, *ADA*, *JAK3*, *IL7RG* genes based on flow cytometry results; chronic granulomatous disease (CGD) were tested for *CYBA*, *NCF1*, *NCF2* genes guided by the results of flow cytometry for the defective intracellular proteins; very early onset inflammatory bowel disease (VEO-IBD) tested for *IL10RA* and *IL10RB*, and leucocyte adhesion deficiency (LAD) patients with defective CD18 expression were tested for *ITGB2* gene.

**Fig. 1:**
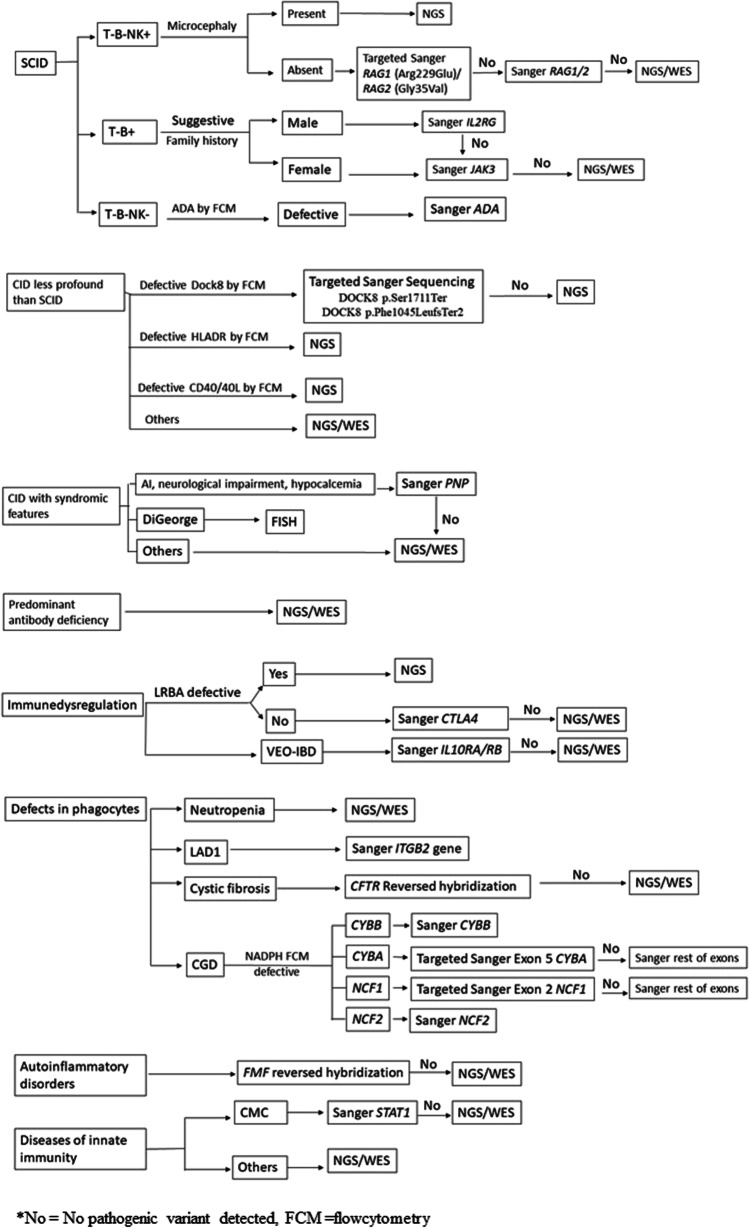
The followed algorithm for genetic testing of IEI according to patients’ phenotypes

Meanwhile, for patients with atypical phenotypes and for those with distinct phenotype, however, Sanger sequencing is not applicable (e.g., large genes), or in case of absence of pathogenic variant in the targeted candidate gene tested by Sanger; NGS or WES was done based on the circumstantial test availability (Fig. 1). For Sanger sequencing, amplification was carried out by polymerase chain reaction (PCR) using designed primers targeting all the exons and exon–intron junctions. PCR products were sequenced on Applied Biosystems™ 3500 Genetic Analyzer (Applied Biosystems, USA) utilizing the same primers used for PCR fragment amplification. Sequences were compared with the reference sequence published by the National Centre for Biotechnology Information and analyzed using the Basic Local Alignment Search Tool (BLAST).

Meanwhile, for next-generation sequencing (NGS), the DNA was used to perform targeted gene capture using a custom capture kit. The panel was designed to cover the exons, exon–intron junctions, and UTRs.

For whole-exome sequencing (WES), Agilent’s SureSelect All Exon V6 + UTR kit was used on a HiSeq4000 system (Illumina, San Diego, CA). The raw reads were first cleaned by removing adapter sequences, trimming low-quality ends, and filtering reads with low quality (Phred quality < 20). The high-quality reads were aligned with the human genome (GRCh37) using Bowtie2 (version 2.3.2, Johns Hopkins University, Baltimore).

Prediction of functional effects of amino acid substitutions was performed using the Polymorphism Phenotyping version 2 software tool (PolyPhen-2) and sorting intolerant from tolerant (SIFT) software. The clinical significance of reported variants and the genotype–phenotype correlation were assessed by protein variation effect analyzer (PROVEAN), VARSOME, and clinically relevant variant (ClinVar) database. Accordingly, the variants were labeled as pathogenic/likely pathogenic/variant of uncertain significance (VUS)/likely benign/benign.

## Results

Among 1000 patients with specific phenotype/probable diagnosis of IEI, a group of 483 Familial Mediterranean fever (FMF) patients and 13 cystic fibrosis patients were diagnosed by strip hybridization assay method and excluded from the study as these patients were not followed up at the PID clinic. Results of only 504 IEI patients are discussed hereby (Supplemental Table 1).

Genetic diagnosis was reached in 282/504 patients (56%) from 246 families: 200 consanguineous (81.3%) and 46 non-consanguineous families (18.7%). We could not detect pathogenic variants that can explain the clinical phenotype in the remaining 222 patients (44%). The diagnosed patients were 171 males (60.6%) including 30 patients with XL-IEI and 111 females (39.4%).

**Fig. 2:**
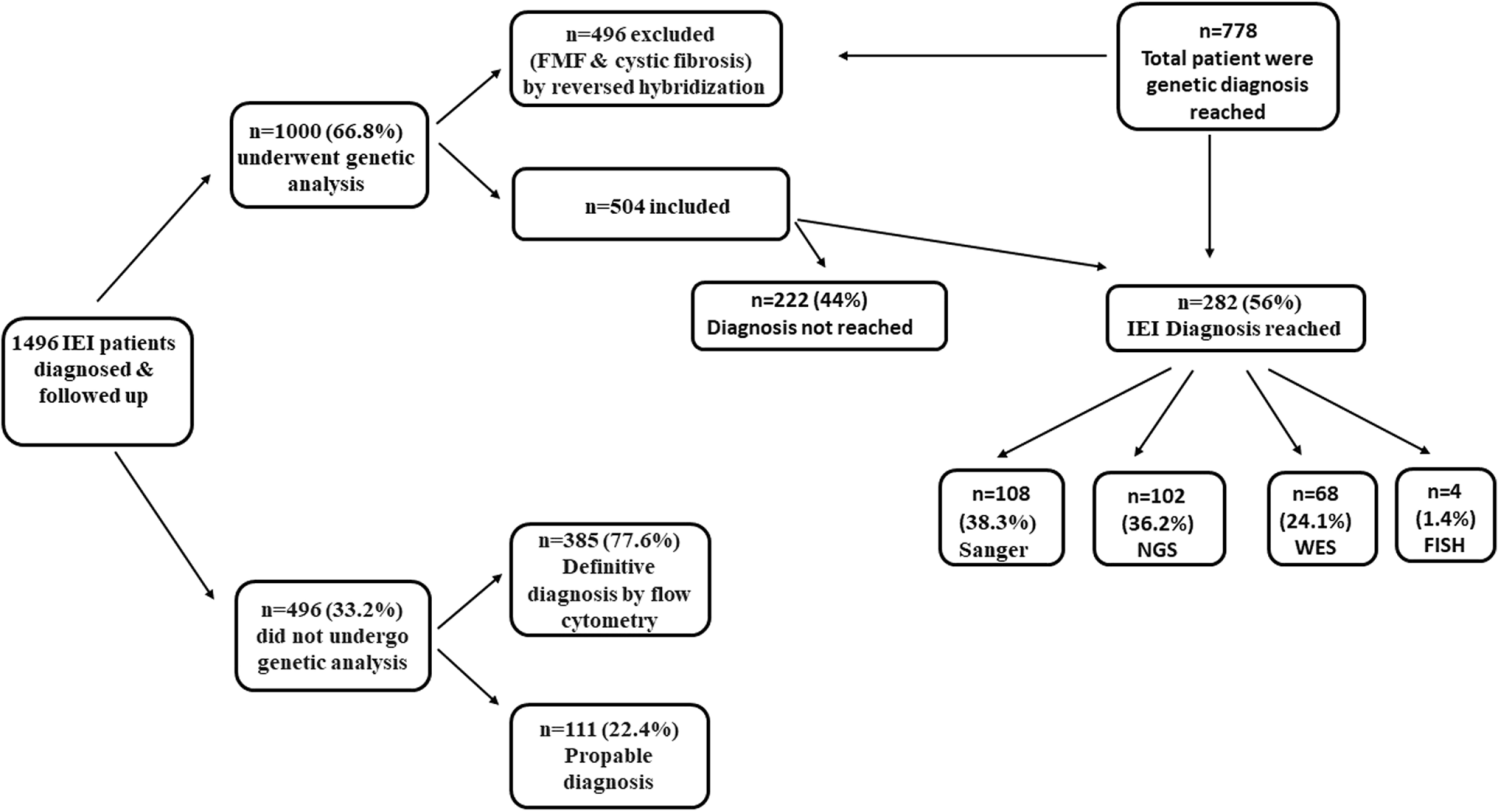
An algorithm representing the total number of IEI patients followed up in Cairo University Children Hospital, the percentage of patients subjected for genetic diagnosis, and the different molecular techniques used to reach definitive diagnosis

Variants were identified by Sanger sequencing in 108/282 patients (38.3%), NGS in 102/282 patients (36.2%), and WES in 68/282 patients (24.1%). Fluorescent in situ hybridization (FISH) was used for the diagnosis of 4 patients (1.4%) with DiGeorge syndrome. A total of 231 patients were tested to begin with by Sanger sequencing with a diagnostic yield of 46.8% (108/231), 282 were tested by NGS/WES with a diagnostic yield of 60.3% (170/282), including 13 patients who were tested by NGS/WES following the failure of Sanger sequencing to identify a pathogenic variant in tested gene (Fig. 2).

**Fig. 3:**
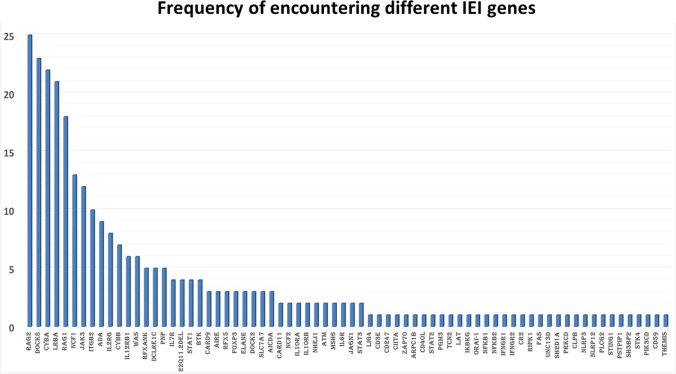
The genetic makeup of IEI patients and the frequency of encountering each gene

Mutations were identified in 72 different genes; *RAG1/2*, *DOCK8*, *CYBA*, *LRBA*, *NCF1*, and *JAK3* genes were the most encountered being detected in 43, 24, 22, 21, 13, and 12 patients respectively (Fig. 3).

Two hundred thirty-three patients, 233/282 (82.6%), inherited the mutations in an autosomal recessive (AR) pattern with 6 patients having two affected genes simultaneously, 32/282 patients (11.3%) in an X-linked (XL) pattern while 18/282 patients (6.4%) had an autosomal dominant (AD) disease. Six patients had hits in two different IEI genes; this included the following: three patients with disease causing variants in both *DOCK8* gene and *CARD9* gene, 1 patient with pathogenic variant in *DOCK8* gene associated with compound heterozygous variants in *AIRE* gene, 2 patients with pathogenic variants in *LRBA* gene and associated variant in *MSH6* gene. One patient had pathogenic variants in two genes with different modes of inheritance: *HUWE1* gene (XL) and *PLCG2* gene (AD). Another six unrelated patients were found to carry the same two simultaneous homozygous variants in *RAG2* gene p.Thr215Ile and p.Arg229Gln. Finally, a patient was diagnosed with two homozygous pathogenic variants: one causing congenital adrenal hyperplasia (non- IEI) and the other in *RFXANK* gene causing MHCII deficiency.

A total of 194 variants were identified in the patients which included 90 missense, 33 nonsense (stop gained), 29 frameshift deletion, 17 intronic, 10 exon deletion, 10 insertion, 4 in frame deletion, 1 insertion deletion variants. One hundred forty-one pathogenic or likely pathogenic variants (72.7%), 44 variants of uncertain significance (VUS) (22.7%), and 9 benign or likely benign variants (4.6%) were identified in genes which would explain patients’ phenotypes. The genetic analysis allowed the identification of novel variants in different genes, for example, novel variants were detected in the *BTK* gene (p.His362Leu and p.Lys175Ter), in the *ITGB2* gene (p.Cys459Ter, p.Gly167Val, p.Gln218Ter), and in the *ADA* gene (p.His17Arg, p.Pro55_Thr57del) (Table 1).

**Table 1:** Variants detected in the IEI genes

Gene & Inheritance	No./gender	c.DNA, AA change	No	*Type*	dbSNP	Classification				gnomAD Exomes ƒ =	Method
						Varsome	Provean	SIFT	Polyphen		
I. Immunodeficiency affecting cellular and humoral immunity (*n* = 128)											
a. Severe combined immunodeficiencies (*n* = 87)											
T-B- severe combined immunodeficiency (*n* = 60)											
* RAG1* (AR)	10♂/8♀	c.424C > T, p.Arg142Ter	*n* = 1	*Nonsense*	rs773929270	Pathogenic	NA	NA	NA	0.00000399	Sanger (*n* = 16) NGS (*n* = 1) WES (*n* = 1)
		c.906C > G, p. Asp302Glu	*n* = 1	*Missense*	-	VUS-LP	Neutral	Damaging	0.982	0.00617	
		c.1003 T > C, p.Cys335Arg	*n* = 1	*Missense*	-	VUS	Deleterious	Damaging	1.000	-	
		c. 2434C > T, p. Gln812Ter	*n* = 3	*Nonsense*	-	Pathogenic	NA	NA	NA	-	
		c.1221G > C, p.Gln407His	*n* = 1	*Missense*	-	Likely pathogenic	Neutral	Damaging	0.994	-	
		c.1228C > T, p.Arg410Trp	*n* = 1	*Missense*	rs758288006	Likely pathogenic	Deleterious	Damaging	1.000	0.000012	
		c.1277_1279delAAG, p.Glu425del	*n* = 1	*Deletion In frame*	-	VUS	Deleterious	NA	NA	-	
		c.1669G > A, p.Ala557Thr	*n* = 1	*Missense*	-	VUS	Neutral	Damaging	0.999	-	
		c.1693G > C, p.Ala565Pro	*n* = 1	*Missense*	-	VUS	Deleterious	Damaging	1.000	-	
		c.1677G > C, p.Arg559Ser	*n* = 1	*Missense*	rs199474681	VUS	Deleterious	Damaging	0.995	-	
		c.1766_1769dupACCT, p. Asn591ProfsTer14	*n* = 2	*Insertion*	-	Pathogenic	NA	NA	NA		
		c.2487_2488delGAinsTT, p.Arg829_Lys830delinsSerTer	*n* = 2	*Deletion Insertion*	-	VUS	NA	NA	NA	-	
		c.1861A > G, p. Lys621Glu	*n* = 1	*Missense*	-	Likely pathogenic	Deleterious	Damaging	0.994	-	
		c.2521C > T, p.Arg841Trp	*n* = 1	*Missense*	rs104894287	Likely pathogenic	Deleterious	Damaging	1.000	0.0000359	
		c.2918G > A, p.Arg973His	*n* = 1	*Missense*	rs1384545687	VUS	Neutral	Damaging	0.999	0.00000399	
		c.2924G > A, p.Arg975Gln	*n* = 1	*Missense*	rs1507396647	Pathogenic	Neutral	Damaging	0.998	0.0000439	
		c.2965G > A, p.Asp989Asn	*n* = 1	*Missense*	-	VUS	Neutral	Damaging	0.998	-	
* RAG2*(AR)	11♂/14♀	c.86 T > C, p.Phe29Ser	*n* = 1	*Missense*	-	VUS	Neutral	Damaging	0.994	-	Sanger (*n* = 23) NGS (*n* = 1) WES (*n* = 1)
		c.104G > T, p.Gly35Val	*n* = 11	*Missense*	rs148508754	VUS	Neutral	Damaging	1.000	0.00000398	
		c.283G > A, p.Gly95Arg	*n* = 1	*Missense*	rs36001797	Likely pathogenic	Deleterious	Damaging	1.000	0.0000318	
		c.379A > T, p.Lys127Ter	*n* = 1	*Nonsense*	-	Pathogenic	NA	NA	NA	-	
		c.442C > T, p.Arg148Ter	*n* = 2	*Nonsense*	rs1315729938	Pathogenic	NA	NA	NA	-	
		c.475C > T, p.Arg159Cys	*n* = 1	*Missense*	rs764485070	VUS	Deleterious	Damaging	1.000	0.0000199	
		c.644C > T, p.Thr215Ile	*n* = 6	*Missense*	rs35691292	Benign	Deleterious	Damaging	0.510	0.00329	
		c.686G > A, p.Arg229Gln	*n* = 6	*Missense*	rs121917894	Likely pathogenic	Deleterious	Damaging	0.999	0.00000796	
		c.980 T > A, p.Val327Asp	*n* = 1	*Missense*	-	VUS	Deleterious	Damaging	0.842	-	
		c.1257C > G, p.Cys419Trp	*n* = 1	*Missense*	-	Likely pathogenic	Deleterious	Damaging	0.999	-	
* DCLRE1C* (AR)	4♀ + 1♂	c.1147C > T, p.Arg383Ter	*n* = 1	*Nonsense*	rs752241422	Likely pathogenic	NA	NA	NA	0.00000796	NGS (*n* = 5)
		Deletion exon 5	*n* = 3	*Deletion Exon*	-	Pathogenic	NA	NA	NA	-	
		Deletion exon 6	*n* = 3	*Deletion Exon*	-	Pathogenic	NA	NA	NA	-	
		c.500C > T, p.Thr167Met	*n* = 1	Missense	rs149556109	VUS-LP	Deleterious	Damaging	1.000	0.000014	
		c.1450_1472dup, p.Phe492Glyfs*60	*n* = 1	Insertion	-	Pathogenic	NA	NA	NA	-	
* ADA* (AR)	7♂/2♀	c.50A > G, p.His17Arg	*n* = 1	*Missense*	-	Likely pathogenic	Deleterious	Damaging	1.000	-	Sanger (*n* = 6) NGS (*n* = 2) WES (*n* = 1)
		c.58G > A, p. Gly20Arg	*n* = 1	*Missense*	rs121908724	Likely pathogenic	Deleterious	Damaging	1.000	0	
		c.164_172delCGCTCACCC, p.Pro55_Thr57del	*n* = 1	*Deletion In frame*	-	Likely pathogenic	NA	NA	NA	-	
		c.956_960delAAGAG, p.Glu319GlyfsTer3	*n* = 1	*Deletion*	rs771266745	Pathogenic	NA	NA	NA	0.000119	
		c.218 + 1G > A	*n* = 3	*Intronic*	rs528390681	Pathogenic	NA	NA	NA	0.0000119	
		c.773G > A, p.Arg258Gln	*n* = 2	*Missense*	rs751635016	Pathogenic	Neutral	Tolerated	0.725	0.00000398	
* LIG4* (AR)	1♀	c.832C > T, p.Arg278Cys	*n* = 1	*Missense*	rs574912936	Likely pathogenic	Deleterious	Damaging	1.000	0.000024	NGS (*n* = 1)
* NHEJ1* (AR)	1♂/1♀	c.178-1G > A	*n* = 2	*Intronic*	-	Pathogenic	NA	NA	NA	-	NGS (*n* = 2)
T-B + severe combined immunodeficiency (*n* = 27)											
* IL2RG* (XL)	8♂	c.2 T > C, p.Met1Thr	*n* = 2	*Missense*	rs886041334	Pathogenic	Neutral	Damaging	0.421	-	WES (*n* = 8)
		c.115G > C, p.Asp39His	*n* = 1	*Missense*	-	Pathogenic	Neutral	Damaging	1.000	-	
		c.677G > A, p.Arg226His	*n* = 1	*Missense*	rs869320660	Pathogenic	Deleterious	Damaging	1.000	-	
		c.865C > T, p.Arg289Ter	*n* = 1	*Nonsense*	rs137852508	Pathogenic	NA	NA	NA	-	
		c.924 + 2 T > G	*n* = 1	*Intronic*	-	Pathogenic	NA	NA	NA	-	
		c.545G > A, p.Cys182Tyr	*n* = 1	*Missense*	rs1064794027	Likely pathogenic	Deleterious	Damaging		-	
		NA	*n* = 1								
* JAK3* (AR)	6♂/6♀	c.308G > A, p.Arg103His	*n* = 1	*Missense*	rs774202259	Pathogenic	Deleterious	Damaging	1.000	0.00000398	Sanger (*n* = 1) WES (*n* = 11)
		c.1027G > C, p.Ala343Pro	*n* = 1	*Missense*	-	Likely pathogenic	Neutral	Damaging	0.954	-	
		c.1207C > T, p.Arg403Cys	*n* = 1	*Missense*	rs1257606008	Likely pathogenic	Neutral	Tolerated	0.108	0.00000797	
		c.2164G > A, p.Val722Ile	*n* = 1	*Missense*	rs3213409	Benign	Neutral	Damaging	0.001	0.00862	
		c.1351C > T, p.Arg451Ter	*n* = 1	*Nonsense*	rs267605358	Pathogenic	NA	NA	NA	0.000032	
		c.1374G > A, p.Trp458Ter	*n* = 1	*Nonsense*	rs1467541371	Likely pathogenic	NA	NA	NA	0	
		c.1765G > A, p.Gly589Ser	*n* = 2	*Missense*	rs886039394	Likely pathogenic	Deleterious	Damaging	1.000	0.00000577	
		c.1142 + 1G > A	*n* = 4	*Intronic*	-	Pathogenic	NA	NA	NA	-	
		c.3011_3013delTCT, p.Phe1004del	*n* = 2	*Deletion* *In frame*	-	Likely pathogenic	Deleterious	NA	NA	-	
* CD3ε* (AR)	1♂	c.269 T > A, p.Leu90Ter	*n* = 1	*Nonsense*	-	Likely pathogenic	NA	NA	NA	-	WES (*n* = 1)
* CD247* (AR)	1♂	c.41C > T, p.Ala14Val	*n* = 1	*Missense*	-	VUS	Neutral	Tolerated	0.002	-	WES (*n* = 1)
* IL7RA* (AR)	3♂/1♀	c.482_483delAA, p.Lys161SerfsTer14	*n* = 1	*Deletion*	-	Pathogenic	NA	NA	NA	-	WES (*n* = 4)
		c.394C > T, p.Pro132Ser	*n* = 2	*Missense*	-	Likely pathogenic	Deleterious	Damaging	1.000	0.00000398	
		c.315C > A, p.Ser105Arg	*n* = 1	*Missense*	-	VUS	Neutral	Tolerated	0.997	-	
* LAT* (AR)	1♀	c.355C > T, p.Arg119Ter	*n* = 1	*Nonsense*	rs746082940	Likely pathogenic	NA	NA	NA	0.000012	WES (*n* = 1)
		c.66 + 176C > T	*n* = 1	*Intronic*	rs34282488	VUS	NA	NA	NA	0.00767	
b. Combined immunodeficiencies generally less profound than severe combined immunodeficiency (*n* = 41)											
* DOCK8* (AR)	14♂/9♀	Deletion Exon 1 to 44	*n* = 1	*Deletion Exon*	-	Pathogenic	NA	NA	NA	-	NGS (*n* = 22) WES (*n* = 1)
		Deletion Exon 6 and 7	*n* = 1	*Deletion Exon*	-	Pathogenic	NA	NA	NA	-	
		Deletion Exon 25 to 33	*n* = 1	*Deletion Exon*	-	Pathogenic	NA	NA	NA	-	
		c.709G > T, p.Glu237Ter	*n* = 2	*Nonsense*	-	Pathogenic	NA	NA	NA	-	
		c.949C > T, p.Arg317Ter	*n* = 1	*Nonsense*	rs113432057	Pathogenic	NA	NA	NA	0.00000398	
		c.3037 T > C, p.Phe1013Leu	*n* = 1	*Missense*	rs779343060	VUS	Neutral	Tolerated	0.209	0.0000557	
		c.3460C > T, p.Arg1154Cys	*n* = 1	*Missense*	rs34390308	VUS-LP	Deleterious	Damaging	1.000	0.00086	
		c.3165_3167delCTT, p.Phe1055del	*n* = 2	*Deletion*	-	VUS	Deleterious	NA	NA	-	
		c.3135delT, p.Phe1045LeufsTer2	*n* = 5	*Deletion*	rs748134881	pathogenic	NA	NA	NA	0	
		c.5132C > A, p.Ser1711Ter	*n* = 6	*Nonsense*	rs1554707993	Pathogenic	NA	NA	NA	-	
		c.5864_5866dupAGA, p.Lys1955dup	*n* = 1	*Insertion*	-	Likely pathogenic	NA	NA	NA	-	
		c.4627-1G > C	*n* = 1	*Intronic*	-	Pathogenic	NA	NA	NA	-	
		c.5962-1G > A	*n* = 1	*Intronic*	-	Pathogenic	NA	NA	NA	-	
* RFXANK* (AR)	2♂/3♀	c.247_250delTCAG, p.Ser83LeufsTer6	*n* = 1	*Deletion*	-	Pathogenic	NA	NA	NA	-	Sanger (*n* = 1) WES (*n* = 4)
		c.431 T > C, p.Leu144Pro	*n* = 2	*Missense*	-	VUS	Deleterious	Damaging	1.000	-	
		c.600delG, p.Asn201ThrfsTer3	*n* = 2	*Deletion*	-	Pathogenic	NA	NA	NA	-	
* CIITA* (AR)	1♂	c.929delA, p.Asn310ThrfsTer2	*n* = 1	*Deletion*	-	Likely pathogenic	NA	NA	NA	-	WES (*n* = 1)
* RFX5* (AR)	3♂	c.116 + 1G > A	*n* = 1	*Intronic*	rs972632936	Pathogenic	NA	NA	NA	0.00000398	NGS (*n* = 1) WES (*n* = 2)
		c.455G > T, p.Gly152Val	*n* = 1	*Missense*	rs1043385719	VUS	Deleterious	Damaging	1.000	-	
		c.715C > T, p.Arg239Ter	*n* = 1	*Nonsense*	rs1233130743	Pathogenic	NA	NA	NA	0.00000399	
* ARPC1B* (AR)	1♀	c.91G > T, p.Glu31Ter	*n* = 1	*Nonsense*	-	Pathogenic	NA	NA	NA	-	NGS (*n* = 1)
* CD40L* (XL)	1♂	c.346G > T, p.Gly116Cys	*n* = 1	*Missense*	-	Pathogenic	Deleterious	Damaging	1.000	-	NGS (*n* = 1)
* ZAP70* (AR)	1♀	c.261C > G, p.Tyr87Ter	*n* = 1	*Nonsense*	-	Likely pathogenic	NA	NA	NA	-	NGS (*n* = 1)
* STK4* (AR)	1♂	NA	*n* = 1								WES (*n* = 1)
* CARD11* (AR)	1♂/1♀	c.2839G > A, p.Glu947Lys	*n* = 1	*Missense*	-	Pathogenic	Neutral	Tolerated	0.06	-	NGS (*n* = 2)
		c.1703C > G, p.Pro568Arg	*n* = 1	*Missense*	-	Likely pathogenic	Deleterious	Damaging	1.000	-	
* DOCK2* (AR)	1♂/2♀	c.316dupT, p.Tyr106Leu fsTer35	*n* = 2	*Insertion*	-	Pathogenic	NA	NA	NA	-	NGS (*n* = 3)
		c.2541delC, p.Phe848LeufsTer18	*n* = 1	*Deletion*	-	Likely pathogenic	NA	NA	NA	-	
II. CID with associated or syndromic features (*n* = 24)											
* WAS* (XL)	6♂	c.274-2A > G	*n* = 1	*Intronic*	-	Pathogenic	NA	NA	NA	-	Sanger (*n* = 1) NGS (*n* = 3) WES (*n* = 2)
		c.347_348insT, p.Phe117LeufsTer5	*n* = 1	*Intronic*	-	Pathogenic	NA	NA	NA	-	
		c.1031delC, p.Pro344LeufsTer101	*n* = 1	*Deletion*	-	Pathogenic	NA	NA	NA	-	
		c.177del, p.Gly60GlufsTer16	*n* = 1	*Deletion*	-	Likely Pathogenic	NA	NA	NA	-	
		c.773 + 3_777 + 6delGAGT	*n* = 2	*Intronic*	-	VUS	NA	NA	NA	-	
* PNP* (AR)	3♂/2♀	c.172C > T, p.Arg58Ter	*n* = 1	*Nonsense*	rs104894460	Pathogenic	NA	NA	NA	0.0000438	Sanger (*n* = 3) NGS (*n* = 1) WES (*n* = 1)
		c.452delA, p.Asn151Met fsTer20	*n* = 3	*Deletion*	-	Pathogenic	NA	NA	NA	-	
		c.682G > C, p.Ala228Pro	*n* = 1	*Missense*	-	VUS	Deleterious	Damaging	1.000	-	
DiGeorge	3♂/1♀	Del 22q11.2	*n* = 4	*Deletion*	-	Pathogenic	NA	NA	NA	-	FISH (*n* = 4)
* ATM* (AR)	2♀	c.3894dup, p.Ala1299CysfsTer3	*n* = 1	*Insertion*	rs587781823	Pathogenic	NA	NA	NA	0.00000796	NGS (*n* = 2)
		c.6346delA, p.Ser2116AlafsTer4	*n* = 1	*Deletion*	-	Pathogenic	NA	NA	NA	-	
		c.1388C > G, p.Ala463Glu	*n* = 1	*Missense*	rs1468739528	Likely pathogenic	Neutral	Damaging	1.000	0.00000398	
		c.370A > G, p.Ile124Val	*n* = 1	Missense	rs148590073	Likely pathogenic	Neutral	Tolerated	0.001	0.00181	
* STAT3* (AD LOF)	2♂	c.1909G > A, p.Val637Met	*n* = 1	*Missense*	rs113994139	Pathogenic	Deleterious	Damaging	1.000	-	NGS (*n* = 2)
		c.1703C > A, p.Pro98Thr	*n* = 1	*Missense*	-	VUS	Deleterious	Damaging	0.998	-	
* PGM3* (AR)	1♂	c.975 T > G, p.Asp325Glu	*n* = 1	*Missense*	rs587777415	Pathogenic	Deleterious	Damaging	1.000	-	NGS (*n* = 1)
* TCN2* (AR)	1♀	c.1195C > T, p.Arg399Ter	*n* = 1	*Nonsense*	rs769817524	Pathogenic	NA	NA	NA	0.00000398	WES (*n* = 1)
* IKBKG* (XL)	1♂	deletion Exon 9	*n* = 1	*Deletion Exon*	-	Pathogenic	NA	NA	NA	-	NGS (*n* = 1)
* ORAI-1* (AR)	1♂	NA	*n* = 1		-						NGS (*n* = 1)
* IL6R* (AR)	1♂	c.10_11delGT, p.Val4FsTer128	*n* = 1	*Deletion*	-	Likely pathogenic	NA	NA	NA	-	WES (*n* = 1)
III. Predominantly antibody deficiencies (*n* = 13)											
* BTK* (XL)	4♂	c.82C > T, p.Arg28Cys	*n* = 1	*Missense*	-	Pathogenic	Deleterious	Damaging	1.000	-	NGS (*n* = 4)
		c.1085A > T, p.His362Leu	*n* = 1	*Missense*	-	Likely pathogenic	Deleterious	Damaging	1.000	-	
		c.523A > T, p.Lys175Ter	*n* = 1	*Nonsense*	-	Pathogenic	NA	NA	NA	-	
		c.1697C > G, p.Pro566Arg	*n* = 1	*Missense*	rs1057521814	Likely pathogenic	Deleterious	Damaging	1.000	-	
* AICDA* (AR)	1♂/2♀	c.331G > A, p.Ala111Thr	*n* = 1	*Missense*	-	Likely pathogenic	Deleterious	Damaging	0.997	-	NGS (*n* = 3)
		c.406delT, p.Ile136Ter	*n* = 2	*Deletion*	rs1453843217	Pathogenic	NA	NA	NA	0.00000404	
* MSH6* (AR)	2♂	c.1904G > C, p.Arg635Thr	*n* = 1	*Missense*	-	VUS	Neutral	Damaging	0.254	-	NGS (*n* = 2)
		c.453dupT, p.Thr152TyrfsTer20	*n* = 1	*Insertion*	-	Pathogenic	NA	NA	NA	-	
* PIK3CD* (AD GOF)	1♂	c.3061G > A, p.Glu1021Lys	*n* = 1	*Missense*	rs397518423	Pathogenic	Deleterious	Damaging	0.999	0	NGS (*n* = 1)
* NFKB1* (AD)	1♂	c.2634A > T, p.Arg878Ser	*n* = 1	*Missense*	rs751865962	VUS	Neutral	Tolerated	0.1	0.00000398	NGS (*n* = 1)
* NFKB2* (AD)	1♀	c.2557C > T, p.Arg853Ter	*n* = 1	*Nonsense*	rs397514332	Pathogenic	NA	NA	NA	-	NGS (*n* = 1)
* CR2* (AR)	1♂	c.1676G > A, p.Gly559Glu	*n* = 1	*Missense*	rs143614333	VUS	Deleterious	Damaging	0.999	0.000848	NGS (*n* = 1)
IV. Diseases of immune dysregulation (*n* = 39)											
* LRBA* (AR)	14♂/7♀	Deletion Exon 1–22	*n* = 1	*Deletion Exon*	-	Pathogenic	NA	NA	NA	-	NGS (*n* = 21)
		Deletion Exon 3–37	*n* = 1	*Deletion Exon*	-	pathogenic	NA	NA	NA	-	
		c.491dupT, p.Leu164PhefsTer12	*n* = 2	*Insertion*	-	Pathogenic	NA	NA	NA	-	
		c. 2170A > G, p.Ile724Val	*n* = 1	*Missense*	rs72719663	Benign	Neutral	Tolerated	0.000	0.0212	
		c.2212 C > T, p.Gln738Ter	*n* = 2	*Nonsense*	-	Pathogenic	NA	NA	NA	-	
		c.2368-2A > G	*n* = 1	*Intronic*	-	Pathogenic	NA	NA	NA	-	
		c.2447delC, p.Pro816LeufsTer4	*n* = 1	*Deletion*	-	Pathogenic	NA	NA	NA	-	
		c.3229G > T, p.Glu1077Ter	*n* = 2	*Nonsense*	-	Pathogenic	NA	NA	NA	-	
		c.3286_3287delTT, p.Phe1096LeufsTer3	*n* = 1	*Deletion*	-	Pathogenic	NA	NA	NA	-	
		Deletion Exon35	*n* = 1	*Deletion Exon*	-	Pathogenic	NA	NA	NA	-	
		c.6587delG, p.Arg2196LeufsTer4	*n* = 2	*Deletion*	-	Pathogenic	NA	NA	NA		
		c.6760G > T, p.Glu2254Ter	*n* = 3	*Nonsense*	-	Pathogenic	NA	NA	NA	-	
		c.8335_8336delGA, p.Asp2779Ter	*n* = 3	*Nonsense*	-	Pathogenic	NA	NA	NA	-	
* SLC7A7* (AR)	3♂	c.1381_1384delATCA, p.Ile461GlufsTer57	*n* = 2	*Deletion*	-	Pathogenic	NA	NA	NA	-	WES (*n* = 3)
		c.404delT, p.Ile135MetfsTer35	*n* = 1	*Deletion*	-	Pathogenic	NA	NA	NA	-	
* AIRE* (AR)	3♂	c.47C > T, p.Thr16Met	*n* = 1	*Missense*	rs179363877	Likely pathogenic	Deleterious	Damaging	1.000	-	NGS (*n* = 3)
		c.91delG, p.Val31SerfsTer158	*n* = 1	*Deletion*	rs140965390	Benign	NA	NA	NA	0.00268	
		c.755C > T, p.Pro252Leu	*n* = 1	*Missense*	rs34397615	Benign	Neutral	Tolerated	0.000	0.00259	
		c.274C > T, p.Arg92Trp	*n* = 1	*Missense*	-	Likely pathogenic	Deleterious	Damaging	1.000	-	
* FOXP3* (XL)	3♂	c.61G > C, p.Gly21Arg	*n* = 1	*Missense*	rs782138321	VUS	Neutral	Damaging	1.000	-	WES (*n* = 1) NGS (*n* = 2)
		c.1040G > A, p.Arg347His	*n* = 1	*Missense*	rs1557115786	pathogenic	Deleterious	Damaging	0.993	-	
		c.1190G > A, p.Arg397Gln	*n* = 1	*Missense*	rs1057520529	Likely pathogenic	Deleterious	Damaging	0.997	-	
* IL10RA* (AR)	2♂	c.499 T > C, p.Tyr167His	*n* = 1	*Missense*	-	VUS	Deleterious	Damaging	1.000	-	NGS (*n* = 2)
		c.632C > T, p.Ser211Phe	*n* = 1	*Missense*	rs143645358	VUS	Deleterious	Damaging	1.000	0.00000398	
* IL10RB* (AR)	1♂/1♀	c.610 T > C, p.Trp204Arg	*n* = 1	*Missense*	-	VUS	Deleterious	Tolerated	0.921	0.00000698	NGS (*n* = 2)
		c.627 T > A, p.Cys209Ter	*n* = 1	*Nonsense*	-	Pathogenic	NA	NA	NA	-	
* RIPK1* (AR)	1♀	NA	*n* = 1		-						WES (*n* = 1)
* UNC13D* (AR)	1♂	c.1193C > T, p.Ser398Leu	*n* = 1	*Missense*	rs747756030	VUS	Deleterious	Damaging	1.000	0.00000399	WES (*n* = 1)
* SH2D1A* (XL)	1♂	c.245dupA, p.Asn82LysTer22	*n* = 1	*Insertion*	-	Pathogenic	NA	NA	NA	-	WES (*n* = 1)
FAS	1♂	c.52delT, p.Leu18TyrfsTer10	*n* = 1	*Deletion*	-	Likely pathogenic	NA	NA	NA	-	NGS (*n* = 1)
* PRKCD* (AR)	1♂	c.1013G > A, p.Trp338Ter	*n* = 1	*Nonsense*	-	Pathogenic	NA	NA	NA	-	NGS (*n* = 1)
V. Congenital defects of phagocyte no., function or both (*n* = 60)											
* CYBA* (AR)	8♂/14♀	c.160_161 InsC, p.Tyr54SerfsTer159	*n* = 1	*Insertion*	-	Pathogenic	NA	NA	NA	-	Sanger (*n* = 21) WES (*n* = 1)
		c.295_301delGTGCCCG, p.Val99ProfsTer90	*n* = 20	*Deletion*	-	Pathogenic	NA	NA	NA	-	
		c.383_393delCACTGCTCGCC, p.Gly128AspfsTer81	*n* = 1	*Deletion*	-	Pathogenic	NA	NA	NA	-	
* NCF1* (AR)	7♂/6♀	c.75_76delGT, p.Tyr26HisfsTer26	*n* = 12	*Deletion*	rs4029402	Pathogenic	NA	NA	NA	0.00018	Sanger (*n* = 13)
* NCF2* (AR)	1♂/1♀	c.239 T > C, p.Leu80Pro	*n* = 1	*Missense*	-	VUS	Deleterious	Tolerated	0.996	-	Sanger (*n* = 2)
		c.574C > T, p.Gln192Ter	*n* = 1	*Nonsense*	-	Pathogenic	NA	NA	NA	-	
* CYBB* (XL)	7♂	c.337 + 1G > A	*n* = 1	*Intronic*	-	Pathogenic	NA	NA	NA	-	Sanger (*n* = 7)
		c.271C > T, p.Arg91Ter	*n* = 1	*Nonsense*	rs886041192	Pathogenic	NA	NA	NA	-	
		c.359 T > C, p.Leu120Pro	*n* = 1	*Missense*	-	Likely pathogenic	Deleterious	Damaging	1.000	-	
		c.1139G > A, p.Trp380Ter	*n* = 1	*Nonsense*	rs1602183244	Pathogenic	NA	NA	NA	-	
		Deletion Exon 2	*n* = 1	*Deletion Exon*	-	Pathogenic	NA	NA	NA	-	
		c.1598_1600delGAG, p.Gly533del	*n* = 2	*Deletion In frame*	-	Likely pathogenic	Deleterious	NA	NA	-	
* ITGB2* (AR)	4♂/6♀	c.1377C > A, p.Cys459Ter	*n* = 1	*Nonsense*	-	Pathogenic	NA	NA	NA	-	Sanger (*n* = 10)
		c.500G > T, p.Gly167Val	*n* = 1	*Missense*	-	Likely pathogenic	Deleterious	Damaging	1.000	-	
		c.505G > A, p.Gly169Arg	*n* = 3	*Missense*	rs137852612	Likely pathogenic	Deleterious	Damaging	1.000	-	
		c.652C > T, p.Gln218Ter	*n* = 1	*Nonsense*	-	Pathogenic	NA	NA	NA	-	
		c.307delG, p.Val103Ter	*n* = 1	*Nonsense*	-	Likely pathogenic	NA	NA	NA	-	
		c.185G > A, p.Cys62Tyr	*n* = 2	*Missense*	-	VUS-LP	Deleterious	Damaging	0.977	-	
		c.306dup, p.Val103SerfsTer39	*N* = 1	*Insertion*	-	Pathogenic	NA	NA	NA	-	
* ELANE* (AD)	3♀	c.452G > C, p.Cys151Ser	*n* = 1	*Missense*	-	Pathogenic	Deleterious	Damaging	0.998	-	WES (*n* = 3)
		c.640G > A, p.Gly214Arg	*n* = 1	*Missense*	rs137854451	Pathogenic	Deleterious	Damaging	1.000	-	
		c.607G > C, p.Gly203Arg	*n* = 1	*Missense*	-	Likely pathogenic	Deleterious	Damaging	1.000	-	
* CLPB*	1♀	c.2099-2100delCT, p.Pro700ArgfsTer22	*n* = 1	*Deletion*	-	Pathogenic	NA	NA	NA	-	WES (*n* = 1)
* JAGN1* (AR)	1♂/1♀	NA	*n* = 2		-						WES (*n* = 2)
VI. Defects in intrinsic and innate immunity (*n* = 16)											
* IL12RB1* (AR)	5♂/1♀	c.1791 + 2 T > G	*n* = 1	*Intronic*	rs554063682	Pathogenic	NA	NA	NA	0.000111	WES (*n* = 6)
		c.643C > T, p.Arg215Trp	*n* = 1	*Missense*	Rs750667928	VUS	Deleterious	Damaging	0.72	0.00000806	
		c.64 + 2 T > G	*n* = 4	*Intronic*	rs765825621	Pathogenic	NA	NA	NA	0.00000416	
* STAT1* (AD GOF)	3♂/1♀	c.862A > C, p.Thr288Pro	*n* = 1	*Missense*	-	Pathogenic	Deleterious	Damaging	0.283	-	Sanger (*n* = 4)
		c.1154C > T, p.Thr385Met	*n* = 1	*Missense*	rs587777630	Pathogenic	Deleterious	Damaging	1.000	-	
		c.1198C > G, p.Leu400Val	*n* = 1	*Missense*	-	VUS	Neutral	Damaging	1.000	-	
		c.1199 T > A, p.Leu400Gln	*n* = 1	*Missense*	-	VUS	Deleterious	Damaging	1.000	-	
* STAT2* (AR)	1♀	c.512A > T, p.Asp171Val	*n* = 1	*Missense*	-	VUS	Deleterious	Damaging	1.000	-	WES (*n* = 1)
* IFNGR2* (AR)	1♂	c.371C > T, p.Ser124Phe	*n* = 1	*Missense*	-	VUS	Deleterious	Damaging	1.000	-	WES (*n* = 1)
* IFNGR1* (AR)	1♀	NA	*n* = 1								WES (*n* = 1)
* CARD9* (AR)	2♂./1♀	c.1546C > T, p.Arg516Trp	*n* = 1	*Missense*	rs773889930	VUS	Neutral	Damaging	0.978	-	NGS (*n* = 3)
		c.442C > T, p.Arg148Trp	*n* = 1	*Missense*	rs149206311	VUS	Deleterious	Tolerated	0.088	0.0000811	
		c.1442-5G > C	*n* = 1	*Intronic*	rs553995269	VUS	NA	NA	NA	0.0000129	
		c.807 + 8C > T	*n* = 1	*Intronic*	rs372934669	Likely benign	NA	NA	NA	0.0000228	
VII. Auto-inflammatory disorders (*n* = 6)											
* NLRP3* (AD)	1♂	c.584C > T, p.Thr195Met	*n* = 1	*Missense*	rs76291085	Likely benign	Neutral	Tolerated	0.001	0.0000358	NGS (*n* = 1)
* NLRP12* (AD)	1♀	c.209G > A, p.Trp70Ter	*n* = 1	*Nonsense*	-	Pathogenic	NA	NA	NA	-	NGS (*n* = 1)
* PLCG2* (AD)	1♂	c.886delT, p.Ser296HisfsTer19	*n* = 1	*Deletion*	-	Pathogenic	NA	NA	NA	-	NGS (*n* = 1)
* STING1* (AD)	1♂	c.575G > T, p.Gly192Val	*n* = 1	*Missense*	rs201096097	Benign	Neutral	Tolerated	0.970	0.000266	NGS (*n* = 1)
* PSTPIP1* (AD)	1♂	c.59C > T, p.Thr20Met	*n* = 1	*Missense*	rs553718554	Benign	Neutral	Damaging	0.616	0.000102	NGS (*n* = 1)
* SH3BP2* (AD)	1♂	c.1103delC, p.Pro368GlnfsTer21	*n* = 1	*Deletion*	-	Pathogenic	NA	NA	NA	-	NGS (*n* = 1)
VIII. Complement deficiencies (*n* = 1)											
* CD59* (AR)	1♂	c.80delA, p.Gln27ArgfsTer53	*n* = 1	*Deletion*	-	Likely pathogenic	NA	NA	NA	-	WES (*n* = 1)
Others											
* THEMIS*	1♀	c.1004C > A, p.Pro335His	*n* = 1	*Missense*	-	VUS-LP	Deleterious	Damaging	0.981	-	WES (*n* = 1)

*VUS* variant of uncertain significance, *VUS-LP* variant of uncertain significance-likely pathogenic, *NA* not available, *NGS* next-generation sequencing, *WES* whole-exome sequencing

**Fig. 4:**
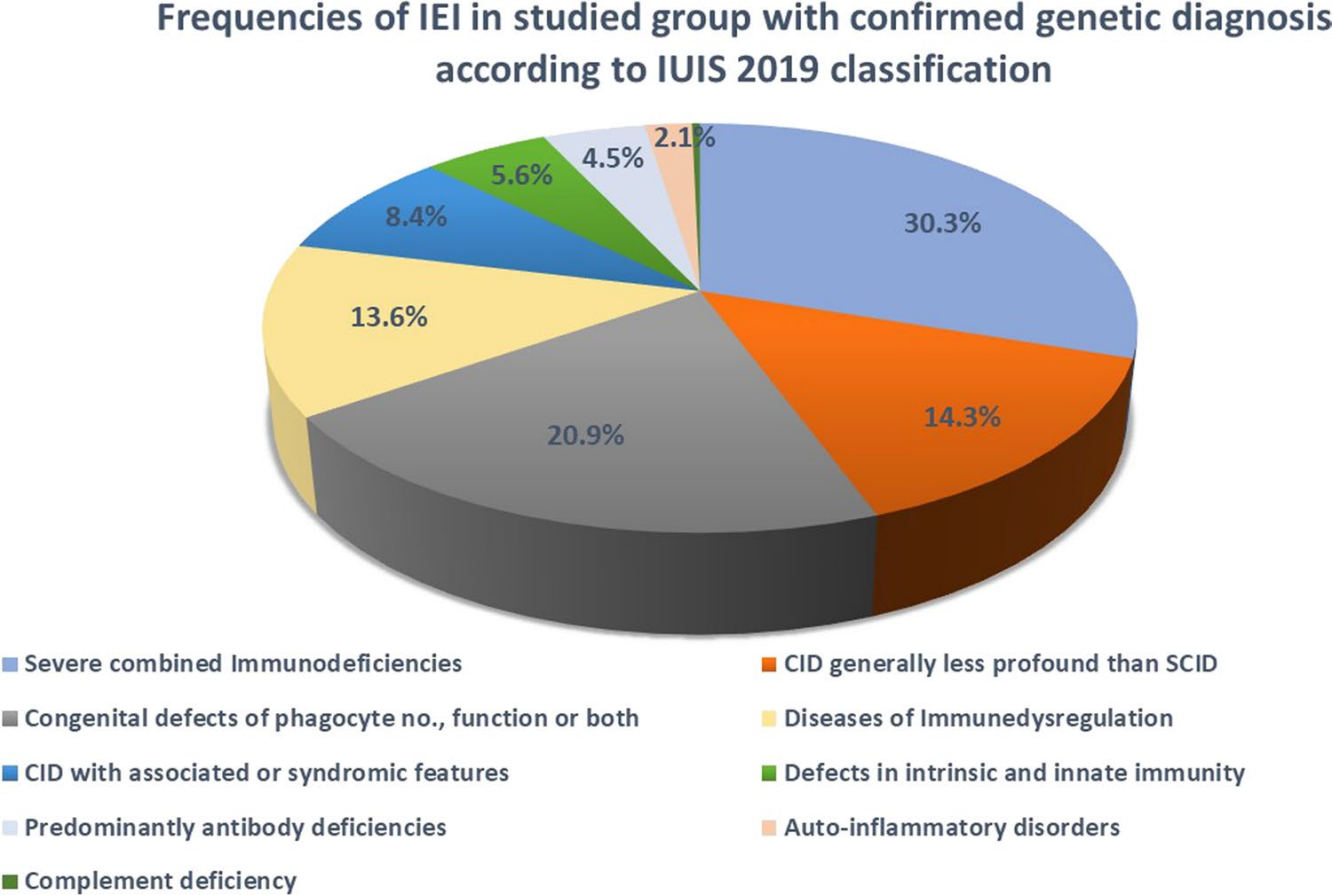
The frequencies of different IEI subgroups in the studied patients with confirmed genetic diagnosis according to the IUIS 2019 classification

Based on the IUIS classification, the most common IEI subgroups within our cohort were immunodeficiency affecting cellular and humoral immunity (*n* = 128, 44.6%) (SCID (*n* = 87, 30.3%) and combined immunodeficiency (CID) generally less profound than SCID (*n* = 41, 14.3%)); congenital defects in the phagocytes (*n* = 60, 20.9%); diseases of immune dysregulation (*n* = 39, 13.6%); CID with associated or syndromic features (*n* = 24, 8.4%); defects in intrinsic and innate immunity (*n* = 16, 5.6%); predominant antibody deficiencies (*n* = 13, 4.5%); auto-inflammatory disorders other than FMF (*n* = 6, 2.1%); and finally complement deficiency was the least common (*n* = 1, 0.4%); it is worth noting a pathogenic variant in *THEMIS* gene that is not present in the IUIS classification 2019 [4] nor in its update in 2021 [6] was identified in one patient (Fig. 4).

Genetic diagnosis was performed for 87 patients with SCID immunophenotyping. In T-B-NK + SCID/Omenn syndrome phenotype without microcephaly, variants in *RAG1/2* were detected in 41 patients from 38 different families. Thirty-nine patients were diagnosed by Sanger sequencing out of 47 patients screened (82.9%) and two patients were diagnosed by NGS; pathogenic variants were detected by NGS in *DCLERIC* gene in 5 patients from 4 families including one patient with atypical phenotype who was diagnosed at the age of 17 years old. Three patients with T-B-NK + phenotype with microcephaly were diagnosed by NGS: *NHEJ1* variant was detected in 2 siblings from the same family and *LIG4* in another patient. Meanwhile, in T-B-NK- phenotype, *ADA* variants were detected in 9 patients from 8 different families.

Regarding the T-B + NK- phenotype, variants in *IL2RG* were identified in 8 male patients and *JAK3* variants in 12 patients from 11 families. While for T-B + NK + phenotype, variants in *IL7RA* were detected in 4 patients from 3 families, *CD247*, *CD3E*, and *LAT* variants in one patient each. In two atypical patients with T-B + NK + phenotype, pathogenic variants in *RAG1* and *RAG2* genes were detected by WES.

Genetic diagnosis was performed for 41 patients with CID generally less profound than SCID: *DOCK8* variants were identified in 23 patients originating from 18 different families (3 of which had associated *CARD9* variants, and one had associated *AIRE* variants). Nine patients were diagnosed with MHCII deficiency having pathogenic variants in *RFXANK* in 5 patients from 4 families, *RFX5* in 3 patients, and *CIITA* in 1 patient. Other genetic variants were identified for CID patients in *DOCK2* gene in 3 patients from 2 families, one of them was previously reported [7]; *CARD11* gene in 2 patients; *Zap70*, *CD40L*, and *ARPC1B* in 1 patient each.

Genetic diagnosis was done for 24 patients with CID with syndromic defects: *WAS* gene variants were detected in 6 patients from 5 different families; 4 patients with DiGeorge syndrome were confirmed by FISH. *PNP* variants were identified in 5 patients from 3 different families. Other rare genetic diagnosis including *ATM*, *STAT3(AD LOF)* were detected in 2 patients each while variants in *PGM3*, *TCN2*, *IKBKG*, *ORAI-1*, and *IL6R* were observed in one patient each.

Genetic diagnosis was done for 13 patients among the group with predominant antibody deficiencies. Pathogenic *BTK* variants were confirmed in 4 symptomatic male patients with defective *BTK* expression by flow cytometry while having normal B cell count and/or near normal immunoglobulin levels. Variants in *AICDA* gene was identified in 3 patients, and other rare variants in *PIK3CD*, *NFKB1*, *NFKB2*, *CR2* were found in 1 patient each. WES in 2 unrelated patients revealed pathogenic/VUS variants in *MSH6* gene associated with pathogenic variants in *LRBA* gene.

Genetic diagnosis was done for 39 patients presenting with immune dysregulation disorders. Twenty-one patients from 15 different consanguineous families had variants in *LRBA* gene (2 patients had associated *MSH6* variants). Other less common genetic diagnosis found were variants in *IL10RA* and *IL10RB* in 4 patients; *FOXP3* gene variants in 3 unrelated male patients; *AIRE* gene variants in 3 unrelated patients (in one of them an associated homozygous pathogenic *DOCK8* gene variant was present); *SLC7A7* gene variants in 3 patients from 2 different families; and *UNC13D*, *PRKCD*, *SH2D1A*, *RIPK1*, *FAS* variants were found in 1 patient each.

Genetic diagnosis was done for 60 patients with congenital defects of phagocyte number, function, or both. CGD was the most common phagocytic function defect in Egypt, Sanger sequencing for only 44 patients was done and revealed variants in *CYBA* in 22 patients from 19 different families, *NCF1* in 13 patients from 9 different families, *CYBB* in 7 male patients from 6 families, *NCF2* in 2 patients. LAD I was the second common functional defect and Sanger sequencing for 10 patients confirmed the presence of pathogenic variants in *ITGB2* gene. While for patients with defects in the phagocytic counts, NGS/WES revealed variants in *ELANE* gene in 3 patients, *JANGN1* variants in 2 siblings from the same family, and *CLPB* in 1 patient.

Genetic diagnosis was done for 16 patients with defects in the intrinsic or innate immunity. Among patients with chronic mucocutaneous candidiasis phenotype, gain-of-function variants in *STAT1* gene were identified in 4 patients. *CARD9* variants were detected in 3 patients; all of them had associated pathogenic *DOCK8* variants, while for patients with a clinical suspension of Mendelian susceptibility to mycobacterial disease, 3 variants in *IL12RB1* were found in 6 patients from 6 different families, a homozygous variant in *IFNGR1* gene causing complete deficiency in 1 patients, a homozygous variant in *IFNGR2* gene causing partial deficiency in 1 patients, and a variant in *STAT2* in 1 patient.

Genetic diagnosis was done for 6 patients with autoinflammatory disorders other than FMF. A pathogenic variant was detected in *PLCG2* gene in 1 patient together with an associated pathogenic variant in *HUWE1* gene. Variants in each of *NLRP3*, *NLRP12*, *STING1*, *PSTPIP1*, *SH3BP2* genes were found in 1 patient each. A summary of genetic results in relation to patients’ phenotypes and preliminary laboratory results is presented in Table 2.

**Table 2:** A summary of genetic results based on clinical diagnosis and preliminary laboratory tests

Clinical diagnosis	Preliminary test	Genes	Genetic test
Omenn syndrome (*n* = 8)	Lymphocyte enumeration, RTE, immunoglobulin level	*RAG1*	Sanger (*n* = 5)
		*RAG2*	Sanger (*n* = 3)
SCID (*n* = 76)	Lymphocyte enumeration, RTE, immunoglobulin level T & B cell subpopulations when indicated, ADA, CD127 expression when suspected	*RAG1*	Sanger (*n* = 10), WES (*n* = 1)
		*RAG2*	Sanger (*n* = 20), WES (*n* = 1)
		*DCLRE1C*	NGS (*n* = 4)
		*ADA*	Sanger (*n* = 6), NGS (*n* = 2), WES (*n* = 1)
		*LAT*	WES (*n* = 1)
		*JAK3*	Sanger (*n* = 1), WES (*n* = 11)
		*IL2RG*	WES (*n* = 8)
		*IL7RA*	WES (*n* = 4)
		*CD247*	WES (*n* = 1)
		*CD3E*	WES (*n* = 1)
		*DOCK2*	WES (*n* = 1)
		*THEMIS*	WES (*n* = 1)
		*PNP*	NGS *n* = 1, WES *n* = 1
Atypical SCID/CID (*n* = 7)	Lymphocyte enumeration, RTE, immunoglobulin level T & B cell subpopulations	*RAG1*	Sanger (*n* = 1), NGS (*n* = 1)
		*RAG2*	Sanger (*n* = 1)
		*NHEJ1*	NGS (*n* = 1)
		*RFXANK*	WES (*n* = 2)
		*RFX5*	WES (*n* = 1)
SCID/Atypical SCID with Microcephaly (*n* = 2)	Lymphocyte enumeration, immunoglobulin level, BTK expression	*LIG4*	NGS (*n* = 1)
		*NHEJ1*	NGS (*n* = 1)
DiGeorge syndrome (*n* = 4)	Lymphocyte enumeration	*Del 22q11.2*	FISH (*n* = 4)
CID/Immune dysregulation (*n* = 2)	Lymphocyte enumeration, Tregs, double negative TCRαβ T cells	*DCLRE1C*	NGS (*n* = 1)
		*SH3BP2*	NGS (*n* = 1)
CID/HIES (*n* = 23)	Lymphocyte enumeration, B cells differentiation, immunoglobulin level, Defective DOCK8 expression	*DOCK8*	NGS (*n* = 23)
HIES (*n* = 4)	Lymphocyte enumeration, B cells differentiation, immunoglobulin level, Normal DOCK8 expression	*STAT3*	NGS (*n* = 2)
		*IL6R*	WES (*n* = 1)
		*LRBA*	NGS (*n* = 1)
HIGM/CID (*n* = 4)	Lymphocyte enumeration, B cells differentiation, immunoglobulin level, CD40/CD40L expression	*CD40LG*	NGS (*n* = 1)
		*AICDA*	NGS (*n* = 3)
CID (*n* = 23)	Lymphocyte enumeration, RTE, HLADR expression	*RFXANK*	WES (*n* = 2)
		*RFX5*	NGS (*n* = 1), WES (*n* = 1)
		*CIITA*	WES (*n* = 1)
	Lymphocyte enumeration, RTE, T cell differentiation, LRBA expression when suspected	*DOCK2*	NGS (*n* = 1), WES (*n* = 1)
		*Zap70*	WES (*n* = 1)
		*ARPC1B*	NGS (*n* = 1)
		*STK4*	WES (*n* = 1)
		*WAS*	Sanger (*n* = 1)
		*ATM*	NGS (*n* = 2)
		*ORAI 1*	NGS (*n* = 1)
		*PNP*	Sanger (*n* = 1)
		*STAT2*	WES (*n* = 1)
		*PSTPIP1*	NGS (*n* = 1)
		*CARD11*	NGS (*n* = 2)
		*CD59*	WES (*n* = 1)
		*CR2*	NGS (*n* = 1)
		*LRBA*	NGS (*n* = 2)
WAS (*n* = 6)	Lymphocyte enumeration, immunoglobulin level, WASP expression, platelet count, mean platelet volume	*WAS*	NGS (*n* = 3), WES (*n* = 2)
		*PGM3*	NGS (*n* = 1)
MSMD (*n* = 7)	Lymphocyte enumeration, DHR	*IL12RB1*	WES (*n* = 5)
		*IFNGR1*	WES (*n* = 1)
		*IFNGR2*	WES (*n* = 1)
CMC (*n* = 6)	Lymphocyte enumeration, DHR	*STAT1*	Sanger (*n* = 4)
		*SLC7A7*	WES (*n* = 2)
LAD (*n* = 10)	CD18/CD11b expression	*ITGB2*	Sanger (*n* = 10)
CGD (*n* = 44)	Defective DHR with bimodal maternal pattern	*CYBB*	Sanger (*n* = 7)
	Defective DHR, FCM defective CYBA	*CYBA*	Sanger (*n* = 21), WES (*n* = 1)
	Defective DHR, FCM defective NCF1	*NCF1*	Sanger (*n* = 13)
	Defective DHR, FCM defective NCF2	*NCF2*	Sanger (*n* = 2)
Neutropenia (*n* = 6)	CBC, Lymphocyte enumeration, anti-neutrophil antibodies	*ELANE*	WES (*n* = 3)
		*JAGN1*	WES (*n* = 2)
		*CLPB*	WES (*n* = 1)
Bone marrow failure syndrome (*n* = 1)	Lymphocyte enumeration	*TCN2*	WES (*n* = 1)
XLA (*n* = 4)	Lymphocyte enumeration, immunoglobulin level, BTK expression	*BTK*	NGS (*n* = 4)
Immune dysregulation (*n* = 11)	Lymphocyte enumeration, Tregs enumeration	*IKBKG*	NGS (*n* = 1)
		*NFKB1*	NGS (*n* = 1)
		*PIK3CD*	NGS (*n* = 1)
		*STING1*	NGS (*n* = 1)
		*PLCG2*	NGS (*n* = 1)
		*NLRP12*	NGS (*n* = 1)
		*LRBA*	NGS (*n* = 5)
EO-IBD (*n* = 5)	Lymphocyte enumeration, B cell differentiation	*IL10RA*	NGS (*n* = 2)
		*IL10RB*	NGS (*n* = 2)
		*RIPK1*	NGS (*n* = 1)
IBD (*n* = 1)	Lymphocyte enumeration, LRBA expression	*LRBA*	NGS (*n* = 1)
CVID (*n* = 3)	Lymphocyte enumeration, B cell differentiation, immunoglobulin level	*NFKB2*	NGS (*n* = 1)
		*LRBA*	NGS (*n* = 2)
APECD (*n* = 2)	Lymphocyte enumeration, Tregs	*AIRE*	NGS (*n* = 2)
IPEX (*n* = 3)	Lymphocyte enumeration, Tregs	*FOXP3*	NGS (*n* = 2), WES (*n* = 1)
IPEX like (*n* = 3)	Lymphocyte enumeration, Tregs, LRBA expression	*LRBA*	NGS (*n* = 3)
Evans syndrome (*n* = 1)	Lymphocyte enumeration	*LRBA*	NGS (*n* = 1)
Autoinflammatory disorder (*n* = 1)	Lymphocyte enumeration	*NLRP3*	NGS (*n* = 1)
ORAI (*n* = 1)	Lymphocyte enumeration, DHR	*SLC7A7*	NGS (*n* = 1)
Lymphoproliferative disorder (*n* = 1)	Lymphocyte enumeration, double negative TCRαβ T cells	*SH2D1A*	NGS (*n* = 1)
ALPS like (*n* = 2)	Lymphocyte enumeration, double negative TCRαβ T cells	*IL12RB1*	WES (*n* = 1)
		*UNC13D*	WES (*n* = 1)
ALPS (*n* = 5)	Lymphocyte enumeration, double negative TCRαβ T cells	*FAS*	NGS (*n* = 1)
		*PRKCD*	NGS (*n* = 1)
		*LRBA*	NGS (*n* = 3)
Screened patients (*n* = 6)	Tested according to affected proband phenotype		Sanger (*n* = 3), NGS (*n* = 3)

*ALPS* autoimmune lymphoproliferative syndrome, *APECD* autoimmune polyendocrinopathy-candidiasis-ectodermal dystrophy, *CBC* complete blood count, *CID* combined immunodeficiency, *CGD* chronic granulomatous disease, *CMC*, chronic mucocutaneous candidiasis, *CVID* common variable immune deficiency, *DHR* dihydrorhodamine test, *EO-IBD* early onset inflammatory bowel disease, *FCM* flow cytometry, *FISH* fluorescent in situ hybridization, *HIGM* hyper-IgM syndrome, *HIES* hyper-IgE syndrome, *IBD* inflammatory bowel disease, *IPEX* immunodysregulation polyendocrinopathy enteropathy X-linked, *LAD* leukocyte adhesions deficiency, *MSMD* Mendelian susceptibility to mycobacterial diseases, *NGS* next-generation sequencing, *RTE* recent thymic emigrants, *SCID* severe combined immunodeficiency, *WAS* Wiskott-Aldrich syndrome, *WES* whole-exome sequencing, *XLA* X-linked agammaglobulinemia

**Fig. 5:**
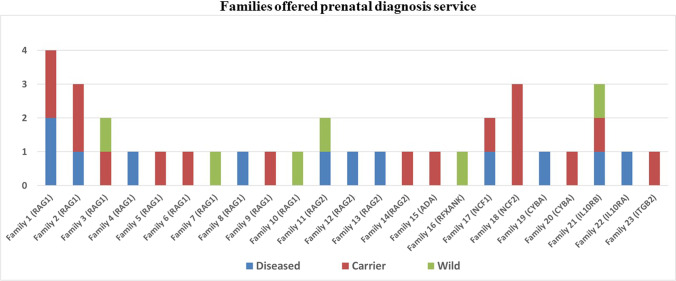
Bar chart representing the different families with known molecular diagnosis of IEI who asked for prenatal diagnosis service and the genetic result of the examined fetuses

Prenatal diagnosis (PND) was offered to 23 families in 35 pregnancies. Fetuses were diagnosed with homozygous pathogenic variants in 12 occasions (34.3%), wild genotype in 6 occasions (17.1%), and heterozygous pathogenic variants in 17 occasions (48.6%). When a heterozygous state was found (as an AR variant was tested), the maternal contamination of the samples was ruled out by means of comparing maternal human leukocyte antigen (HLA) typing with that of the chorionic villous samples. The pregnancies with normal or carrier fetuses were continued, while those with diseased fetuses were dealt with according to the families’ decisions after proper counselling (Fig. 5).

## Discussion

The genetic heterogeneity of the IEI as well as the delay in the diagnosis in atypical cases leads to significant morbidity and mortality. Establishing definitive genetic diagnosis is very important for patients’ management [8].

In 2016, Cairo University PID center had published its own 5 years of experience (2010–2014) in this field being one of the largest tertiary referral centers that receive patients from Egypt as well as from nearby Arab countries. The study helped to identify the spectrum and different patterns of IEI in Egyptian children; however, the genetic diagnosis was only available for 102 patients (22.26%) of the clinically diagnosed patients (*n* = 476) [9]. With the increased utilization of genetic testing and widening of the collaborative efforts with different centers, we were able to reach the genetic diagnosis for 778 patients out of 1496 patients diagnosed and followed up till 2021 (52%). It is worth noting that due to the increased experience with flow cytometry in our center, a definitive diagnosis was reached for many IEI phenotypes without the need for molecular testing for an additional number of patients 385/1496 (25.7%). The functional tests using flow cytometry in our center had been validated by the results of genetic testing in previous studies. This is very evident in CGD, LAD I, *WAS*, *BTK*, *DOCK8*, *MHC II*, and *LRBA* deficiency patients; thus, the need for genetics in these patients is limited to families who request prenatal diagnosis in future pregnancies [10–13]. As expected for a highly consanguineous population, most patients suffered from AR IEI disorders (82.6%), whereas XL recessive and AD modes of inheritance were less frequently encountered. This agrees with previous studies conducted on similar populations from Middle East and North Africa (MENA) [14], and disagrees with a study on 160 Chinese patients utilizing targeted NGS were the diagnostic yield was 43.8% with majority of patients having X-linked disease [15].

In our cohort, the most common IEI subgroups were immunodeficiency affecting cellular and humoral immunity (44.6%) followed by congenital defects in the phagocytes (20.9%), diseases of immune dysregulation (13.6%), CID with associated or syndromic features (8.4%), while defects in intrinsic and innate immunity and predominantly antibody deficiencies were much less common. In a recent study published about IEI from MENA region, the genetic approach was performing targeted genetic sequencing based on the phenotype. WES was done for patients in whom targeted sequencing was not diagnostic or if their clinical phenotypes resemble several genetic defects. Their diagnostic yield was 83% and the highest diagnosis was predominantly antibody deficiency [14].

In contrast to our findings, a study presenting one of the largest molecular studies from India showed that defects in intrinsic and innate immunity, diseases of immune dysregulation, and antibody deficiencies were the most common PIDs in their studied cohort. Their diagnostic yield was 42%, less than ours, and their molecular testing was done by Sanger sequencing and NGS targeting a customized panel [3].

WES testing of 350 PID Iranian patients revealed pathogenic/likely pathogenic variants in 35% of the cases, a percent higher than the results we obtained from our WES analysis although our overall yield is higher (55.2%) [16]. This is probably because of the genetic approach adopted in our center in which WES is done only in cases where targeted sequencing fails to reach the diagnosis.

In a study about the efficacy of NGS versus WES on a large PID cohort, Platt and his colleagues had a diagnostic yield close to ours (56%), yet they concluded that WES has advantage of lower cost than NGS. However, this approach can not be applied in many countries with limited genetic facilities [17].

Seventy-one genes (15.5%) out of the IEI known genes were identified in Egyptian patients, which highlights the importance of discovering the genetic makeup for each disease phenotype in each country. One patient had pathogenic variant in *THEMIS* gene which is not present in the IUIS 2019 classification nor in its update in 2021; thus, more extensive functional studies for this gene are highly recommended.

Sanger sequencing was used to identify the genetic defects in 108 patients (38.3%) mainly when patients presented with classical phenotype and in genes with small number of exons. For example, in T-B-NK + SCID/Omenn syndrome phenotype without microcephaly (*n* = 47), Sanger sequencing of *RAG1/2* genes identified the pathogenic variants in 39 patients (82.9%), thus limiting the need for NGS to almost less than one-fifth of the patients with these phenotypes.

In T-B + SCID patients (*n* = 47), WES helped in the diagnosis of 26 patients with variants in *ILRG* and *JAK3* in 19 patients with T-B + phenotype. Based on these results, Sanger sequencing for *JAK3/IL2RG* (based on patient’s gender, family history, and immunophenotype) will help in diagnosis of nearly 73% of these patients thus decreasing the need for NGS/WES to no more than 27% of patients in this group [18].

The molecular diagnosis helped to identify variants that were repeatedly detected in several patients from different families in the same gene and even being detected mainly in certain geographic distribution within the country. Target screening for these hot spots first—when utilizing Sanger sequencing—saved time and decreased the cost [12]. For example in T-B-NK + SCID patients, Gly35Val variant in *RAG2* gene had been identified in 11 patients coming from three governorates from North Egypt, while the p.Thr215Ile variant and p.Arg229Gln had been detected together both in homozygous form in 6 patients mostly coming from upper Egypt. In *DOCK8* deficiency, p.Ser1711Ter and p.Phe1045LeufsTer2 were identified in 11 patients (47.8% of genetically diagnosed *DOCK8* patients). In CGD/*NCF1* patients, only one variant, p.Tyr26HisfsTer26, had been identified in all patients and in CGD/*CYBA*, one variant, p.Val99ProfsTer90, had been detected in almost all patients. These two variants were also the only ones reported by another study from upper Egypt [19].

The molecular diagnosis elaborates double genetic affection in two independent genes in a few patients (*n* = 8). Three patients had pathogenic *DOCK8* and VUS *CARD9* variants. Two had extensive fungal infections to which they were receiving antifungal treatment. The third was diagnosed early being screened as a sibling of an index case and was transplanted once diagnosed.

Two patients with pathogenic *LRBA* variants had associated variants (one pathogenic, one VUS) in *MSH6* gene. Both patients were referred to a specialist for investigating the probability of having autosomal recessive mismatch repair cancer syndrome. These coincidences might need more extensive studies on the probable cause for this linkage and at times may require adjustment of therapeutic modalities for some of the patients.

One patient had pathogenic *DOCK8* and 2 heterozygous *AIRE* variants; one was previously published as disease-causing variant [20] and the other was a frameshift variant (disease causing by mutation taster); the patient had low T regs by flow cytometry. The patient was kept under close supervision for autoimmune manifestations. One patient had *RFXANK* pathogenic variant associated with a pathogenic variant in *CYP21A2* gene causing congenital adrenal hyperplasia; thus, a specialist was involved in the patient management plan. One patient had a pathogenic variant in *PLCG2* gene together with pathogenic variant in *HUWE1* gene; this patient had delayed mental and motor milestones.

In some cases, the molecular testing may reveal a genetic diagnosis that was not expected by the patient’s phenotype; for instance, two patients presented with classical T-B + SCID phenotype underwent WES; interestingly, the first patient showed a homozygous variant in *RAG1* gene and the other showed a homozygous variant in *RAG2* gene [21]. A male patient presented with autoimmune lymphoproliferative syndrome phenotype and had 20% CD4-CD8-TCRαβ + cells, WES revealed homozygous pathogenic variant in *IL12RB1*.

A global genetic sequencing pilot program offered by Jeffrey Modell Foundation to identify specific IEI defects leads to alteration in disease management in 40% of patients [22]. Examples for changing the treatment modality based on molecular diagnosis was a SCID male infant with T-B-NK- phenotype, the molecular diagnosis of adenosine deaminase deficiency allowed the clinician to instantly start polyethylene-glycol-conjugated bovine adenosine deaminase (PEG-ADA), and in the absence of an HLA matched sibling donor, the patient was treated with autologous hematopoietic stem cell gene therapy (HSC-GT) for the correction of his immunodeficiency [23].

Patients with VEO-IBD are usually subjected to a precision medicine approach to choose between stem cell transplantation, antibiotics, abatacept therapy, or other therapies [24]. One of our VEO-IBD patients was diagnosed by NGS with homozygous variant in *IL10RA* gene; once diagnosis was reached, the patient was subjected to SCT from a completely matched sibling.

It is important to note that the evaluation of different variants pathogenicity is critical to formulate clinically reliable results. Despite the advances in computing technology, this process cannot be fully automated and still requires clinical and expertise judgment [25]. In the current study, we reported on 9 benign/likely benign variants by Varsome, however predicted to be pathogenic by other computational analysis, previously published in patients with similar phenotypes [20, 26] and/or had minor allele frequency. This highlights the importance of functional validation of variants whenever possible.

Identification of the pathogenic variants allowed to offer genetic counselling service and PND for many families. In 2017, PND was done in 12 pregnancies from 10 different families with testing only available for 5 genes initially (*RAG1*, *RAG2*, *NCF1*, *NCF2*, *IL10RB*) [27]. But with the growing experience and the increase in the number of genes available for Sanger sequencing in our center (5 more genes were added: *ADA*, *CYBA*, *RFXANK*, *IL10RA*, *ITGB2*), PND was performed in 35 pregnancies from 23 families. This helped these families choose among different available options.

It is worth noting that outlining the incidence of IEI in Egyptian patients is the basis in understanding the disease phenotype and is a nidus for building up a national Egyptian registry.

## Conclusion

Molecular diagnosis of IEI patients is highly important specially in atypical phenotypes as genetic heterogeneity of the diseases and the delay in diagnosis leads to high morbidity and mortality. It helps in offering genetic counseling for many families and taking the best therapeutic decisions for the patients. Studying the candidate genes in each disease phenotype may help to find a founder pathogenic variant, a hot gene exon, and linked genetic inheritance, and validate the results of other easier, less expensive diagnostic tests. In many developing countries, genetic testing cost is still high or even unavailable; thus, each center should follow its own algorithm to select the patients that benefit most from genetic testing and the families who need definitive diagnosis. Collaboration and networking with other centers help in achieving genetic diagnosis and adjusting personalized treatment for many patients. Finally, studying genetic background of IEI patients from consanguineous populations helps better understand the molecular immunopathogenesis of IEI diseases.

## Supplementary Information

Below is the link to the electronic supplementary material.

Supplementary file1 (DOCX 163 KB)

## Data Availability

The authors confirm that data supporting the findings of the study are available in the article. Raw data were generated in Cairo University Specialized Children Hospital. The datasets generated during and/or analyzed during the current study are available from the corresponding author on reasonable request.
