# Supplementary material for: Genetic Testing in Egyptian Patients with Inborn Errors of Immunity: a Single-Center Experience
Source: J Clin Immunol. 2022 Apr 28;42(5):1051–70. doi: 10.1007/s10875-022-01272-y (PMC9402523; doi:10.1007/s10875-022-01272-y)
Supplement: Supplementary file 1 — Supplementary file1 (DOCX 163 KB) [file 10875_2022_1272_MOESM1_ESM.docx]

**Supplemental Table 1: Patients demographic data, clinical phenotype, laboratory and genetic tests results**

| Kindred | Consanguinity | Proband No. | Gender | Clinical phenotype | Flow cytometry | Genetic test | Gene | Inheritance | Variant detected | Reference |
| --- | --- | --- | --- | --- | --- | --- | --- | --- | --- | --- |
| Severe Combined Immunodeficiencies | | | | | | | | | | |
| T-B- Severe Combined Immunodeficiency | | | | | | | | | | |
| 1 | Yes | 1 | F | Omenn | Low CD19 | Sanger | *RAG1* | AR | Homo c.424C>T, p.Arg142Ter | 21 |
| 2 | Yes | 2 | M | Omenn | CD4 lymphopenia, Low CD19 | Sanger | *RAG1* | AR | Homo c.1277_1279delAAG, p.Glu425del | 21,26 |
| 3 | Yes | 3 | M | Omenn | Low CD19 | Sanger | *RAG1* | AR | Homo c.1677G>C, p.Arg559Ser | 21 |
| 4 | No | 4 | F | Omenn | low CD19 | Sanger | *RAG1* | AR | Homo c.2434C >T, p.Gln812Ter | 21,26 |
| 5 | Yes | 5 | F | Omenn | Low CD19 | Sanger | *RAG1* | AR | Homo c.2924G >A, p.Arg975Gln | 21,26 |
| 6 | Yes | 6 | F | SCID | T-B-NK+ | Sanger | *RAG1* | AR | Hetero c.906C>G, p.Asp302Glu,  Hetero c.1669G>A, p.Ala557Thr |  |
| 7 | No | 7 | F | Atypical SCID/ CID | CD4 lymphopenia, Low CD19 | Sanger | *RAG1* | AR | Hetero c.1003T>C, p.Cys335Arg  Hetero c.2434C>T, p.Gln812Ter | 21 |
| 8 | Yes | 8 | M | SCID | T-B-NK+ | Sanger | *RAG1* | AR | Homo c.1221G>C, p.Gln407His | 21 |
| 9 | Yes | 9 | F | SCID | T-B-NK+ | Sanger | *RAG1* | AR | Homo c.1228C>T, p.Arg410Trp |  |
| 10 | Yes | 10 | M | SCID | T-B-NK+ | Sanger | *RAG1* | AR | Homo c.1693G>C, p.Ala565Pro | 21 |
| 11 | Yes | 11^a^ | M | SCID | T-B-NK+ | Sanger | *RAG1* | AR | Homo c.1766_1769dupACCT, p.Asn591ProfsTer14 | 21 |
|  |  | 12^b^ | M | SCID | T-B-NK+ | Sanger | *RAG1* | AR | Homo c.1766_1769dupACCT, p.Asn591ProfsTer14 | 21 |
| 12 | Yes | 13 | M | SCID | T-B-NK+ | Sanger | *RAG1* | AR | Homo c.2434C >T, p.Gln812Ter |  |
| 13 | Yes | 14 | F | SCD | T-B-NK+ | Sanger | *RAG1* | AR | Homo c.2487_2488delGAinsTT‎, p.Arg829_Lys830delinsSerTer | 21,26 |
| 14 | Yes | 15 | M | SCID | Low CD19 | Sanger | *RAG1* | AR | Hetero c.2487_2488delGAinsTT, p.Arg829_Lys830delinsSerTer,  Hetero c.1861 A>G, p.Lys621Glu | 21 |
| 15 | Yes | 16 | F | SCID | T-B+NK+ | WES | *RAG1* | AR | Homo c.2521C>T, p.Arg841Trp | 21 |
| 16 | No | 17 | M | Atypical SCID/ CID | Low CD3, CD4 lymphopenia | NGS | *RAG1* | AR | Homo c.2918G>A, p.Arg973His | 21 |
| 17 | Yes | 18 | M | SCID | T-B-NK+ | Sanger | *RAG1* | AR | Homo c.2965G>A, p.Asp989Asn |  |
| 18 | Yes | 19 | F | SCID | T-B-NK+ | Sanger | *RAG2* | AR | Homo c.86T>C, p.Phe29Ser | 21 |
| 19 | Yes | 20 | F | SCID | T-B-NK+ | Sanger | *RAG2* | AR | Homo c.104G>T, p.Gly35Val | 21 |
| 20 | Yes | 21 | F | SCID | T-B-NK+ | Sanger | *RAG2* | AR | Homo c.104G>T, p.Gly35Val | 21 |
| 21 | Yes | 22 | M | SCID | T-B-NK+ | Sanger | *RAG2* | AR | Homo c.104G>T, p.Gly35Val | 21 |
| 22 | Yes | 23 | M | SCID | T-B-NK+ | Sanger | *RAG2* | AR | Homo c.104G>T, p.Gly35Val | 21 |
| 23 | Yes | 24 | M | SCID | T-B-NK+ | Sanger | *RAG2* | AR | Homo c.104G>T, p.Gly35Val | 21 |
| 24 | Yes | 25 | F | SCID | T-B-NK+ | Sanger | *RAG2* | AR | Homo c.104G>T, p.Gly35Val |  |
| 25 | Yes | 26^a^ | M | SCID | T-B-NK+ | Sanger | *RAG2* | AR | Homo c.104G>T, p.Gly35Val |  |
|  |  | 27^b^ | M | SCID | T-B-NK+ | Sanger | *RAG2* | AR | Homo c.104G>T, p.Gly35Val |  |
| 26 | Yes | 28 | M | SCID | T-B-NK+ | Sanger | *RAG2* | AR | Homo c.104G>T, p.Gly35Val |  |
| 27 | Yes | 29 | F | SCID | T-B-NK+ | Sanger | *RAG2* | AR | Homo c.104G>T, p.Gly35Val |  |
| 28 | Yes | 30 | F | SCID | T-B-NK+ | Sanger | *RAG2* | AR | Homo c.104G>T, p.Gly35Val |  |
| 29 | Yes | 31 | F | SCID | T-B-NK+ | Sanger | *RAG2* | AR | Homo c.283G>A, p.Gly95Arg | 21 |
| 30 | No | 32 | F | Atypical SCID/ CID | T-B-NK+ | Sanger | *RAG2* | AR | Homo c.379A>T, p.Lys127Ter | 21 |
| 31 | Yes | 33^a^ | M | Omenn | Low CD19 | Sanger | *RAG2* | AR | Homo c.442C>T, p.Arg148Ter | 21 |
|  |  | 34^b^ | F | SCID turned to Omenn | T-B-NK+ | Sanger | *RAG2* | AR | Homo c.442C>T, p.Arg148Ter | 21 |
| 32 | Yes | 35 | F | Omenn | Low CD19 | Sanger | *RAG2* | AR | Homo c.475C>T, p.Arg159Cys | 21 |
| 33 | Yes | 36^a^ | M | SCID | T-B-NK+ | Sanger | *RAG2* | AR | Homo c.644C>T, p.Thr215Ile,  Homo c.686G>A, p.Arg229Gln | 21,26 |
|  |  | 37^b^ | M | SCID | T-B-NK+ | Sanger | *RAG2* | AR | Homo c.644C>T, p.Thr215Ile,  Homo c.686G>A, p.Arg229Gln | 21 |
| 34 | Yes | 38 | F | SCID | T-B-NK+ | NGS | *RAG2* | AR | Homo c.644C>T, p.Thr215Ile,  Homo c.686G>A, p.Arg229Gln | 21 |
| 35 | No | 39 | F | SCID | T-B-NK+ | Sanger | *RAG2* | AR | Homo c.644C>T, p.Thr215Ile,  Homo c.686G>A, p.Arg229Gln | 21 |
| 36 | Yes | 40 | M | SCID | T-B-NK+ | Sanger | *RAG2* | AR | Homo c.644C>T, p.Thr215Ile,  Homo c.686G>A, p.Arg229Gln | 21 |
| 37 | Yes | 41 | M | SCID | T-B-NK+ | Sanger | *RAG2* | AR | Homo c.644C>T, p.Thr215Ile,  Homo c.686G>A, p.Arg229Gln |  |
| 38 | Yes | 42 | F | SCID | T-B+NK+ | Sanger | *IL7RA* | AR | No pathogenic variant | 21 |
|  |  |  |  |  |  | WES | *RAG2* | AR | Homo c.980T>A, p.Val327Asp |  |
| 39 | Yes | 43 | F | Omenn | Low CD19 | Sanger | *RAG2* | AR | Homo c.1257C>G, p.Cys419Trp | 21 |
| 40 | Yes | 44 | F | SCID | T-B-NK+ | NGS | *DCLRE1C* | AR | Homo c.1147C>T, p.Arg383Ter |  |
| 41 | Yes | 45^a^ | F | SCID | T-B-NK+ | Sanger | *RAG1/2* | AR | No pathogenic variant |  |
|  |  |  |  |  |  | NGS | *DCLRE1C* | AR | Hetero Deletion exon 5, Hetero Deletion exon 6 |  |
|  |  | 46^b^ | F | SCID | T-B-NK+ | NGS | *DCLRE1C* | AR | Hetero Deletion exon 5, Hetero Deletion exon 6 |  |
| 42 | Yes | 47 | F | SCID | T-B-NK+ | NGS | *DCLRE1C* | AR | Hetero Deletion exon 5, Hetero Deletion exon 6 |  |
| 43 | No | 48 | M | CID/Immune dysregulation | CD4 lymphopenia, Low CD19 | NGS | *DCLRE1C* | AR | Heteroc.500C>T p.Thr167Met  Hetero c.1450_1472dup, p.Phe492Glyfs*60 |  |
| 44 | Yes | 49 | F | SCID | T-B-NK- | WES | *ADA* | AR | Homo c.50A>G, p.His17Arg |  |
| 45 | Yes | 50 | M | SCID | T-B-NK- | Sanger | *ADA* | AR | Homo c.58G>A, p. Gly20Arg | 23 |
| 46 | Yes | 51 | M | SCID | T-B-NK- | Sanger | *ADA* | AR | Homo c.164_172delCGCTCACCC, p.Pro55_Thr57del |  |
| 47 | Yes | 52 | M | SCID | T-B-NK- | Sanger | *ADA* | AR | Homo c.956_960delAAGAG, p.Glu319GlyFsTer3 |  |
| 48 | No | 53 | M | SCID | T-B-NK- | Sanger | *ADA* | AR | Homo c.218+1G>A |  |
| 49 | Yes | 54^a^ | M | SCID | T-B-NK- | NGS | *ADA* | AR | Homo c.218+1 G>A |  |
|  |  | 55^b^ | F | SCID | T-B-NK+ | NGS | *ADA* | AR | Homo c.218+1 G>A |  |
| 50 | Yes | 56 | M | SCID | T-B-NK-/Marked lymphopenia | Sanger | *ADA* | AR | Homo c.773G>A, p.Arg258Gln |  |
| 51 | Yes | 57 | M | SCID | T-B-NK- | Sanger | *ADA* | AR | Homo c.773G>A, p.Arg258Gln |  |
| 52 | Yes | 58 | F | SCID/ Microcephaly | T-B-NK+ | NGS | *LIG4* | AR | Homo c.832C>T, p.Arg278Cys |  |
| 53 | Yes | 59 | F | Atypical SCID/ Microcephaly | Lymphopenia/ Low CD19 | NGS | *NHEJ1* | AR | Homo c.178-1G>A |  |
|  |  | 60 | M | Atypical SCID/CID | Low CD19 | NGS | *NHEJ1* | AR | Homo c.178-1G>A |  |
| 54 | Yes | 61 | F | SCID | T-B+NK- | WES | *LAT* | AR | Hetero c.355C>T, p.Arg119*, Hetero c.66+176C>T |  |
| T- B+ Severe combined immunodeficiency | | | | | | | | | | |
| 55 | Yes | 62 | F | SCID | T-B+NK- | WES | *JAK3* | AR | Homo c.308G>A, p.Arg103His | 18 |
| 56 | Yes | 63 | F | SCID | T-B+NK- | WES | *JAK3* | AR | Homo c.1027G>C, p.Ala343Pro | 18 |
| 57 | Yes | 64 | M | SCID | T-B+NK- | WES | *JAK3* | AR | Homo c.1207C>T , p.Arg403Cys,  Homo c.2164G>A, p.Val722Ile | 18 |
| 58 | Yes | 65 | M | SCID | T-B+NK+ | Sanger | *IL7RA* | AR | No pathogenic variant | 18 |
|  |  |  |  |  |  | WES | *JAK3* | AR | Homo c.1351C>T, p.Arg451Ter |  |
| 59 | Yes | 66 | F | SCID | T-B+NK- | WES | *JAK3* | AR | Homo c.1374G>A, p.Trp458Ter | 18 |
| 60 | Yes | 67 | F | SCID | T-B+NK- | WES | *JAK3* | AR | Homo c.1765G>A, p.Gly589Ser | 18 |
| 61 | Yes | 68 | F | SCID | T-B+NK- | Sanger | *JAK3* | AR | Hetero c.1765G>A, p.Gly589Ser,  Hetero c.1142+1G>A | 18 |
| 62 | Yes | 69^a^ | M | SCID | T-B+NK- | WES | *JAK3* | AR | Homo c.1142+1 G>A | 18 |
|  |  | 70^b^ | F | SCID | T-B+NK- | WES | *JAK3* | AR | Homo c.1142+1 G>A | 18 |
| 63 | Yes | 71 | M | SCID | T-B+NK- | WES | *JAK3* | AR | Homo c.1142+1 G>A | 18 |
| 64 | Yes | 72 | M | SCID | T-B+NK- | WES | *JAK3* | AR | Homo c.3011_3013delTCT, p.Phe1004del | 18 |
| 65 | Yes | 73 | M | SCID | T-B+NK- | WES | *JAK3* | AR | Homo c.3011_3013delTCT, p.Phe1004del | 18 |
| 66 | No | 74 | M | SCID | T-B+NK- | WES | *IL2RG* | XL | Hemi c.2T>C, p.Met1Thr | 18 |
| 67 | No | 75 | M | SCID | T-B+NK- | WES | *IL2RG* | XL | Hemi c.2T>C, p.Met1Thr | 18 |
| 68 | No | 76 | M | SCID | T-B+NK- | WES | *IL2RG* | XL | Hemi c.115G>C, p.Asp39His | 18 |
| 69 | No | 77 | M | SCID | T-B+NK- | WES | *IL2RG* | XL | Hemi c.677G>A, p.Arg226His | 18 |
| 70 | Yes | 78 | M | SCID | T-B+NK- | WES | *IL2RG* | XL | Hemi c.865C>T, p.Arg289Ter | 18 |
| 71 | No | 79 | M | SCID | T-B+NK- | WES | *IL2RG* | XL | Hemi c.924+2T>G | 18 |
| 72 | No | 80 | M | SCID | T-B+NK- | WES | *IL2RG* | XL | NA |  |
| 73 | Yes | 81 | M | SCID | T-B+NK- | WES | *IL2RG* | XL | Hemi c.545G>A, p.Cys182Tyr |  |
| 74 | Yes | 82 | M | SCID | T-B+NK+ | WES | *IL7RA* | AR | Homo c.482_483delAA, p.Lys161fsTer14 | 18 |
| 75 | Yes | 83^a^ | F | SCID | T-B+NK+ | WES | *IL7RA* | AR | Homo c.394C>T, p.Pro132Ser |  |
|  |  | 84^b^ | M | SCID | T-B+NK+ | WES | *IL7RA* | AR | Homo c.394C>T, p.Pro132Ser |  |
| 76 | Yes | 85 | M | SCID | T-B+NK+ | WES | *IL7RA* | AR | Homo c.315 C>A, p.Ser105Arg |  |
| 77 | Yes | 86 | M | SCID | T-B+NK+ | WES | *CD247* | AR | Homo c.41C>T, p.Ala14Val |  |
| 78 | Yes | 87 | M | SCID | T-B+NK+ | Sanger | *IL7RA* | AR | No pathogenic variant | 18 |
|  |  |  |  |  |  | WES | *CD3E* | AR | Homo c.269T>A, p.Leu90Ter |  |
| Combined immunodeficiencies generally less profound than Severe Combined Immunodeficiency | | | | | | | | | | |
| 79 | Yes | 88 | F | CID | CD4 lymphopenia, Absent HLADR | WES | *RFXANK* | AR | Homo c.247_250delTCAG, p.Ser83LeufsTer6‎ | 13 |
| 80 | Yes | 89^a^ | F | CID | CD4 lymphopenia, Absent HLADR | WES | *RFXANK* | AR | Homo c.431T>C, p.Leu144pro | 13 |
|  |  | 90^b^ | M | Screened | Absent HLADR | Sanger | *RFXANK* | AR | Homo c.431T>C, p.Leu144pro | 13 |
|  |  |  |  |  |  | RH | *CYP21A2* | AR | NA |  |
| 81 | Yes | 91 | M | Atypical SCID | T-B+NK- | WES | *RFXANK* | AR | Homo c.600delG, p.Asn201ThrfsTer3‎ | 13 |
| 82 | Yes | 92 | F | Atypical SCID | T-B+NK- | WES | *RFXANK* | AR | Homo c.600delG, p.Asn201ThrfsTer3‎ | 13 |
| 83 | Yes | 93 | M | Atypical SCID | T-B+NK+ | WES | *RFX5* | AR | Homo c.116+1G>A | 13 |
| 84 | Yes | 94 | M | CID | CD4 lymphopenia, Absent HLADR | WES | *RFX5* | AR | Homo c.455G>T, p.Gly152Val | 13 |
| 85 | Yes | 95 | M | CID | CD4 lymphopenia | NGS | *RFX5* | AR | Homo c.715C>T, p.Arg239Ter | 13 |
| 86 | Yes | 96 | M | CID | CD4 lymphopenia, Absent HLADR | WES | *CIITA* | AR | Homo c.929delA, p.Asn310ThrfsTer2 | 13 |
| 87 | Yes | 97 | M | CID | Normal immunophenotype | WES | *Zap70* | AR | Homo c.261C>G, p.Tyr87Ter |  |
| 88 | Yes | 98^a^ | F | SCID | T-B+NK+ | Sanger | *IL7RA* | AR | No pathogenic variant |  |
|  |  |  |  |  |  | WES | *DOCK2* | AR | Homo c.316dupT, p.Tyr106LeufsTer35 |  |
|  |  | 99^b^ | F | CID | T-B+NK+ | WES | *DOCK2* | AR | Homo c.316dupT, p.Tyr106LeufsTer35 |  |
| 89 | Yes | 100 | M | CID | CD4 lymphopenia, Low CD19 | Sanger | *RAG1/2* | AR | No pathogenic variant | 7 |
|  |  |  |  |  |  | NGS | *DOCK2* | AR | Homo c.2541delC, p.Phe848LeyFsTer18 |  |
| 90 | Yes | 101 | F | CID/HIES | CD4 lymphopenia, Defective DOCK8, Low memory B cells | NGS | *DOCK8* | AR | Homo Deletion Exon 1 to 44 | 10 |
|  |  |  |  |  |  |  | *CARD9* | AR | Homo c.1546C>T, p.Arg516Trp |  |
| 91 | Yes | 102 | M | CID/HIES | CD4 lymphopenia, Defective DOCK8, Low memory B cells | NGS | *DOCK8* | AR | Homo Deletion Exon 6 and 7 | 10 |
| 92 | Yes | 103 | M | CID/HIES | CD4 lymphopenia, Defective DOCK8, Low memory B cells | NGS | *DOCK8* | AR | Homo Deletion Exon 25 to 33 | 10 |
|  |  |  |  |  |  |  | *CARD9* | AR | Hetero c.1442-5G>C, Hetero c.807+8C>T |  |
| 93 | Yes | 104 | F | CID/HIES | CD4 lymphopenia, Defective DOCK8, Low memory B cells | NGS | *DOCK8* | AR | Homo c.709G>T, p.Glu237Ter | 10 |
| 94 | Yes | 105 | M | CID/HIES | CD4 lymphopenia, Defective DOCK8, Low memory B cells | NGS | *DOCK8* | AR | Homo c.709G>T, p.Glu237Ter | 10 |
| 95 | Yes | 106 | M | CID/HIES | CD4 lymphopenia, Defective DOCK8,Low memory B cells | NGS | *DOCK8* | AR | Homo c.949C>T, p.Arg317Ter |  |
| 96 | Yes | 107 | M | CID/HIES | CD4 lymphopenia, Defective DOCK8, Low memory B cells | NGS | *DOCK8* | AR | Hetero c.3037 T>C, p.Phe1013Leu  Hetero c.3160 C>T, p.Arg1054Cys |  |
| 97 | Yes | 108^a^ | F | CID/HIES | CD4 lymphopenia,  Defective DOCK8 | NGS | *DOCK8* | AR | Homo [c.3165_3167delCTT](https://varsome.com/variant/hg19/DOCK8(NM_203447.3):c.3165_3167delCTT), p.Phe1055del ‎ | 10 |
|  |  | 109^b^ | F | CID/HIES | CD4 lymphopenia, Defective DOCK8, Low memory B cells | NGS | *DOCK8* | AR | Homo [c.3165_3167delCTT](https://varsome.com/variant/hg19/DOCK8(NM_203447.3):c.3165_3167delCTT), p.Phe1055del ‎ | 10 |
| 98 | Yes | 110^a^ | M | CID/HIES | CD4 lymphopenia, Defective DOCK8, Low memory B cells | NGS | *DOCK8* | AR | Homo c.3135delT, p.Phe1045LeufsTer2 | 10 |
|  |  | 111^b^ | M | CID/HIE | CD4 lymphopenia, Defective DOCK8, Low memory B cells | NGS | *DOCK8* | AR | Homo c.3135delT, p.Phe1045LeufsTer2 | 10 |
|  |  | 112^c^ | M | CID/HIES | CD4 lymphopenia, Defective DOCK8, Low memory B cells | NGS | *DOCK8* | AR | Homo c.3135delT, p.Phe1045LeufsTer2 | 10 |
| 99 | Yes | 113^a^ | M | CID/HIES | CD4 lymphopenia, Defective DOCK8, Low memory B cells | NGS | *DOCK8* | AR | Homo c.3135delT, p.Phe1045LeufsTer2 | 10 |
|  |  | 114^b^ | M | CID/HIES | CD4 lymphopenia, Defective DOCK8, Low memory B cells, Low Tregs | NGS | *DOCK8* | AR | Homo c.3135delT, p.Phe1045LeufsTer2 | 10 |
|  |  |  |  |  |  |  | *AIRE* | AR | Hetero c.91delG p.Val31SerfsTer157,  Hetero c.755C>T, p.Pro252Leu |  |
| 100 | Yes | 115 | F | CID/HIES | CD4 lymphopenia, Defective DOCK8, Low memory B cells | NGS | *DOCK8* | AR | Homo c.5132C>A, p.Ser1711Ter | 10 |
| 101 | Yes | 116 | F | CID/HIES | CD4 lymphopenia, Defective DOCK8, Low memory B cells | NGS | *DOCK8* | AR | Homo c.5132C>A, p.Ser1711Ter | 10 |
| 102 | Yes | 117 | M | CID/HIES | CD4 lymphopenia, Defective DOCK8, Low memory B cells | NGS | *DOCK8* | AR | Homo c.5132C>A, p.Ser1711Ter | 10 |
| 103 | Yes | 118^a^ | F | CID/HIES | CD4 lymphopenia, Defective DOCK8, Low memory B cells | NGS | *DOCK8* | AR | Homo c.5132C>A, p.Ser1711Ter |  |
|  |  | 119^b^ | M | CID/HIES | CD4 lymphopenia, Defective DOCK8, Low memory B cells | NGS | *DOCK8* | AR | Homo c.5132C>A, p.Ser1711Ter |  |
|  |  |  |  |  |  |  | *CARD9* | AR | Homo c.442C>T, p.Arg148Trp |  |
| 104 | Yes | 120 | M | CID/HIES | CD4 lymphopenia, Defective DOCK8, Low memory B cells | WES | *DOCK8* | AR | Homo c.5132C>A, p.Ser1711Ter |  |
| 105 | Yes | 121 | M | CID/HIES | CD4 lymphopenia, Normal DOCK8, Low memory B cells | NGS | *DOCK8* | AR | Homo c.5864_5866dupAGA, p.Lys1955dup |  |
| 106 | Yes | 122 | M | CID/HIES | CD4 lymphopenia, Defective DOCK8, Low memory B cells | NGS | *DOCK8* | AR | Homo c.5962-1G>A |  |
| 107 | Yes | 123 | F | CID/HIES | CD4 lymphopenia, Defective DOCK8, Low memory B cells | NGS | *DOCK8* | AR | Homo c.4627-1G>C |  |
| 108 | Yes | 124 | F | CID | CD8 lymphopenia, Normal DOCK8 | NGS | *ARPC1B* | AR | Homo c.91G>T, p.Glu31Ter |  |
| 109 | Yes | 125 | M | HIGM/CID | Normal immunophenotype, Normal CD40, Low CD40L | NGS | *CD40LG* | XL | Hemi c.346G>T, p.Gly116Cys |  |
| 110 | Yes | 126 | M | CID | CD4 lymphopenia | WES | *STK4* | AR | NA |  |
| CID with associated or syndromic features | | | | | | | | | | |
| 111 | No | 127 | M | WAS | Normal immunophenotype | NGS | *WAS* | XL | Hemi c.274-2A>G |  |
| 112 | No | 128 | M | CID | CD4 lymphopenia, Normal RTE, Normal HLADR | Sanger | *WAS* | XL | Hemi c.347_348insT, p.Val105CysfsTer15 |  |
| 113 | Yes | 129 | M | WAS | - | WES | *WAS* | XL | Hemi c.1031delC, p.Pro344LeufsTer101 |  |
| 114 | No | 130 | M | WAS | Low CD19 | WES | *WAS* | XL | Hemi c.177del, p.Gly60GlufsTer16 |  |
| 115 | Yes | 131^a^ | M | WAS | Normal immunophenotype, Defective WASP | NGS | *WAS* | XL | Hemi c.7773+3_777+6delGAGT |  |
|  |  | 132^b^ | M | WAS | CD4 lymphopenia, Defective WASP | NGS | *WAS* | XL | Hemi c.7773+3_777+6delGAGT |  |
| 116 | Yes | 133 | F | CID | Low CD3 | NGS | *ATM* | AR | Homo c.3894dup, p.Ala1299CysfsTer3 |  |
| 117 | No | 134 | F | CID | CD4 lymphopenia | Sanger | *RAG1/2* | AR | No pathogenic variant |  |
|  |  |  |  |  |  | NGS | *ATM* | AR | Hetero c.6346delA, p.Ser2116AlafsTer4  Hetero c.1388C>G, p.Ala463Glu |  |
| 118 | Yes | 135 | M | WAS | Normal WASP | NGS | *PGM3* | AR | Homo c.975T>G, p.Asp325Glu |  |
| 119 | Yes | 136 | M | HIES | Normal immunophenotype,  Normal DOCK8 | NGS | *STAT3* | AD LOF | Hetero c.1909G>A, p.Val637Met |  |
| 120 | Yes | 137 | M | HIES | Normal immunophenotype,  Normal DOCK8 | NGS | *STAT3* | AD LOF | Homo c.1703 C>A, p.Pro98Thr |  |
| 121 | Yes | 138 | M | CID | Low CD19 | NGS | *ORAI 1* | AR | NA |  |
| 122 | Yes | 139 | M | Immune dysregulation | Normal immunophenotype, Low Tregs | NGS | *IKBKG* | XL | Hemi Deletion Exon 9 |  |
| 123 | Yes | 140 | F | Bone marrow failure syndrome | Normal immunophenotype | WES | *TCN2* | AR | Homo c.1195C>T, p.Arg399Ter |  |
| 124 | Yes | 141 | F | SCID | Low CD3 | WES | *PNP* | AR | Homo c.172C>T, p.Arg58Ter |  |
| 125 | Yes | 142^a^ | M | SCID | T-B-NK- | Sanger | *RAG1/2* | AR | No pathogenic variant |  |
|  |  |  |  |  |  | NGS | *PNP* | AR | Homo [c.452delA](https://varsome.com/variant/hg19/PNP(NM_000270.4):c.452delA), p.Asn151MetfsTer20 |  |
|  |  | 143^b^ | F | Screened | Normal immunophenotype | Sanger | *PNP* | AR | Homo [c.452delA](https://varsome.com/variant/hg19/PNP(NM_000270.4):c.452delA), p.Asn151MetfsTer20 |  |
|  |  | 144^c^ | M | Screened | T-B-NK- | Sanger | *PNP* | AR | Homo [c.452delA](https://varsome.com/variant/hg19/PNP(NM_000270.4):c.452delA), p.Asn151MetfsTer20 |  |
| 126 | No | 145 | M | CID | CD4 lymphopenia | Sanger | *PNP* | AR | Homo c.682G>C, p.Ala228Pro |  |
| 127 | No | 146 | M | HIES | Low CD3, Normal DOCK8 | WES | *IL6R* | AR | Homo c.10_11delGT, p.Val4FsTer128 |  |
| 128 | Yes | 147 | M | CID | CD4 lymphopenia, Low cd19 | NGS | *CARD11* | AR | Homo c.1703C>G, p.Pro568Arg |  |
| 129 | Yes | 148 | F | CID | Normal immunophenotype, Normal DOCK8, Normal LRBA | Sanger | *RAG1/2* | AR | No pathogenic variant |  |
|  |  |  |  |  |  | NGS | *CARD11* | AR | Homo c.2839G>A, p.Glu947Lys |  |
| 130 | Yes | 149 | M | DiGeorge syndrome | Normal immunophenotype | FISH |  | AR | Homo Del 22q11.2 |  |
| 131 | No | 150 | M | DiGeorge syndrome | T-B+NK+ | FISH |  | AR | Homo Del 22q11.2 |  |
| 132 | Yes | 151 | F | DiGeorge syndrome | T-B+NK+ | FISH |  | AR | Homo Del 22q11.2 |  |
| 133 | Yes | 152 | M | DiGeorge syndrome | Low CD4 | FISH |  | AR | Homo Del 22q11.2 |  |
| Predominantly antibody deficiencies | | | | | | | | | | |
| 134 | Yes | 153 | M | XLA | Absent CD19 | NGS | *BTK* | XL | Hemi c.82C>T, p. Arg28Cys |  |
| 135 | No | 154 | M | XLA | Absent CD19, Absent BTK | NGS | *BTK* | XL | Hemi 1085A>T, p.His362Leu |  |
| 136 | No | 155 | M | XLA | Absent CD19, Absent BTK | NGS | *BTK* | XL | Hemi c.523A>T, p.Lys175Ter |  |
| 137 | No | 156 | M | XLA | Absent CD19, Absent BTK | NGS | *BTK* | XL | Hemi c.1697C>G, p.Pro566Arg |  |
| 138 | Yes | 157 | F | CVID | Low CD19 | NGS | *NFKB2* | AD | Hetero c.2557C>T, p.Arg853Ter |  |
| 139 | No | 158 | M | Immune dysregulation | Low CD19, Normal LRBA | NGS | *NFKB1* | AD | Hetero c.2634A>T, p.Arg878Ser |  |
| 140 | No | 159 | M | Immune dysregulation | CD4 lymphopenia, Low CD19 | Sanger | *RAG1/2* | AR | No pathogenic variant |  |
|  |  |  |  |  |  | NGS | *PIK3CD* | AD GOF | Hetero c.3061G>A, p.Glu1021Lys |  |
| 141 | Yes | 160 | M | HIGM | Normal immunophenotype | Sanger | *RAG1/2* | AR | No pathogenic variant |  |
|  |  |  |  |  |  | NGS | *AICDA* | AR | Homo c.331G>A, p.Ala111Thr |  |
| 142 | Yes | 161^a^ | F | HIGM | CD4 lymphopenia, Low CD19, Normal CD40 | NGS | *AICDA* | AR | Homo c.406del, p.Ile136Ter |  |
|  |  | 162^b^ | F | HIGM | Low CD19, Normal CD40 | NGS | *AICDA* | AR | Homo c.406del, p.Ile136Ter |  |
| 143 | Yes | 163 | M | CID | CD4 lymphopenia | NGS | *CR2* | AR | Homo c.1676G>A, p.Gly559Glu |  |
| Diseases of Immune dysregulation | | | | | | | | | | |
| 144 | Yes | 164 | M | HLH/ALPS like | Increase Double negative αβ T cells | WES | *UNC13D* | AR | Homo c.1193C>T, p.Ser398Leu |  |
| 145 | Yes | 165 | M | ALPS | Increase Double negative αβ T cells | NGS | *PRKCD* | AR | Homo c.1013G>A, p.Trp338Ter |  |
| 146 | Yes | 166^a^ | M | CMC | Normal immunophenotype | WES | *SLC7A7* | AR | Homo c.1381_1384delATCA, p.Ile461Glu fsTer57 |  |
|  |  | 167^b^ | M | CMC | Normal immunophenotype | WES | *SLC7A7* | AR | Homo c.1381_1384delATCA, p.Ile461Glu fsTer57 |  |
| 147 | Yes | 168 | M | ORAI | Normal immunophenotype & DHR | NGS | *SLC7A7* | AR | Homo c.404delT, p.Ile135MetfsTer35 |  |
| 148 | Yes | 169 | M | APECD | Normal immunophenotype | NGS | *AIRE* | AR | Homo c.47C>T, p.Thr16Met |  |
| 159 | Yes | 170 | M | APECD | Normal immunophenotype,  Low Tregs | NGS | *AIRE* | AR | Homo c.274C>T, p.Arg92Trp |  |
| 150 | Yes | 171 | F | EO-IBD | CD4 lymphopenia, Low CD19 | NGS | *IL10RB* | AR | Homo c.610T>C, p.Trp204Arg |  |
| 151 | Yes | 172 | F | EO-IBD | Normal immunophenotype | NGS | *IL10RB* | AR | Homo c.627T>A, p.Cys209Ter |  |
| 152 | Yes | 173 | M | EO-IBD | Normal immunophenotype | NGS | *IL10 RA* | AR | Homo c.499T>C, p.Tyr167His |  |
| 153 | Yes | 174 | M | EO-IBD | Normal immunophenotype | NGS | *IL10RA* | AR | Homo c.632C>T, p.Ser211Phe |  |
| 154 | Yes | 175 | M | IPEX | Normal immunophenotype,  Low Tregs | NGS | *FOXP3* | XL | Hemi c.61G>C, p.Gly21Arg |  |
| 155 | Yes | 176 | M | IPEX | CD4 lymphopenia, absent Tregs | NGS | *FOXP3* | XL | Hemi c.1190G>A, p.Arg397Gln |  |
| 156 | Yes | 177 | M | IPEX | Normal immunophenotype | WES | *FOXP3* | XL | Hemi c.1040G>A, p.Arg347His |  |
| 157 | Yes | 178 | M | IPEX-like | Normal immunophenotype, Defective LRBA | NGS | *LRBA* | AR | Homo Deletion exon 1-22 | 11 |
| 158 | Yes | 179 | F | IDDM/Idiopathic pulmonary fibrosis | Normal immunophenotype, Defective LRBA | NGS | *LRBA* | AR | Homo Deletion exon 3-37 | 11 |
| 159 | Yes | 180^a^ | M | CVID | Normal immunophenotype, Defective LRBA | NGS | *LRBA* | AR | Homo c.491dupT, p.Leu164PheFsTer12 | 11 |
|  |  | 181^b^ | M | CVID | Normal immunophenotype | NGS | *LRBA* | AR | Homo c.491dupT, p.Leu164PheFsTer12 |  |
| 160 | Yes | 182 | F | HIES | Normal immunophenotype, Normal DOCK8 | NGS | *LRBA* | AR | Homo c.2170A>G, p.Ile724Val | 11 |
| 161 | Yes | 183 | F | Evans syndrome | Normal immunophenotype | NGS | *LRBA* | AR | Homo c.2212C>T, p.Gln738Ter | 11 |
| 162 | Yes | 184 | F | IDDM | Normal immunophenotype | NGS | *LRBA* | AR | Homo c.2212C>T, p.Gln738Ter |  |
| 163 | Yes | 185 | M | CID | Normal immunophenotype, Low LRBA | NGS | *LRBA* | AR | Homo c.2368-2A>G | 11 |
| 164 | Yes | 186 | M | IPEX-like | Normal immunophenotype, Defective LRBA | NGS | *LRBA* | AR | Homo c.2447delC, p.Pro816LeufsTer4 | 11 |
| 165 | Yes | 187^a^ | F | Screened | Normal immunophenotype, Defective LRBA | NGS | *LRBA* | AR | Homo c.3229G>T, p.Glu1077Ter | 11 |
|  |  | 188^b^ | M | ALPS | Normal immunophenotype, Defective LRBA | NGS | *LRBA* | AR | Homo c.3229G>T, p.Glu1077Ter | 11 |
|  |  |  |  |  |  |  | *MSH6* | AR | Homo c.1904G>C, p.Arg635Thr |  |
| 166 | Yes | 189 | M | ALPS/IBD | Normal immunophenotype, Defective LRBA | NGS | *LRBA* | AR | Homo c.3286_3287delTT, p.Phe1096LeufsTer3 | 11 |
|  |  |  |  |  |  |  | *MSH6* | AR | Homo c.453dupT,‎ p.Thr152TyrfsTer20 ‎ |  |
| 167 | Yes | 190 | M | IBD | Normal immunophenotype, Defective LRBA | NGS | *LRBA* | AR | Homo Deletion exon35 | 11 |
| 168 | Yes | 191^a^ | M | ALPS | Low CD19, Defective LRBA | NGS | *LRBA* | AR | Homo c.6587delG, p.Arg2196LeufsTer4 | 11 |
|  |  | 192^b^ | M | Immune dysregulation/CID | Low CD19, Defective LRBA | NGS | *LRBA* | AR | Homo c.6587delG, p.Arg2196LeufsTer4 |  |
| 169 | Yes | 193^a^ | M | IDDM | Normal immunophenotype | NGS | *LRBA* | AR | Homo c.6760G>T, p.Glu2254Ter | 11 |
|  |  | 194^b^ | M | Immune dysregulation | Normal immunophenotype | NGS | *LRBA* | AR | Homo c.6760G>T, p.Glu2254Ter | 11 |
|  |  | 195^c^ | M | Screened | Normal immunophenotype, Defective LRBA | NGS | *LRBA* | AR | Homo c.6760G>T, p.Glu2254Ter | 11 |
| 170 | Yes | 196^a^ | F | Screened | Normal immunophenotype, Defective LRBA | NGS | *LRBA* | AR | Homo c.8334_8336delGA, p.Asp2779Ter | 11 |
|  |  | 197^b^ | M | IBD/IPEX-like | Low CD19, Defective LRBA | NGS | *LRBA* | AR | Homo c.8334_8336delGA, p.Asp2779Ter | 11 |
| 171 | Yes | 198 | M | CID | CD4 lymphopenia, Low CD19, Defective LRBA | Sanger | *RAG1/2* | AR | No pathogenic variant | 11 |
|  |  |  |  |  |  | NGS | *LRBA* | AR | Homo c.8335_8336delGA, p.Asp2779Ter |  |
| 172 | Yes | 199 | F | EO-IBD | Low CD19 | NGS | *RIPK1* | AR | NA |  |
| 173 | Yes | 200 | M | ALPS | Increase Double negative αβ T cells | NGS | *FAS* | AR | Homo c.52delT, p.Leu18TyrfsTer10 ‎ |  |
| 174 | No | 201 | M | Lymphoproliferative disorder | Normal immunophenotype | NGS | *SH2D1A* | XL | Hemi c.245dupA, p.Asn82LysTer22 |  |
| Congenital defects of phagocytes no., function or both | | | | | | | | | | |
| 175 | No | 202 | F | Neutropenia | Normal immunophenotype | WES | *ELANE* | AD | Hetero c.452G>C, p.Cys151Ser |  |
| 176 | No | 203 | F | Neutropenia | Normal immunophenotype | WES | *ELANE* | AD | Hetero c.640G>A, p.Gly214Arg |  |
| 177 | No | 204 | F | Neutropenia | Normal immunophenotype | WES | *ELANE* | AD | Hetero c.607G>C, p.Gly203Arg |  |
| 178 | Yes | 205^a^ | M | Neutropenia | Normal immunophenotype | WES | *JAGN1* | AR | NA |  |
|  |  | 206^b^ | F | Neutropenia | Normal immunophenotype | WES | *JAGN1* | AR | NA |  |
| 179 | Yes | 207 | F | Neutropenia | Normal immunophenotype | WES | *CLPB* | AR | Homo c.2099_2100delCT, p.Pro700ArgfrsTer22 |  |
| 180 | No | 208 | M | CGD | Defective DHR,  Bimodal maternal DHR pattern | Sanger | *CYBB* | XL | Hemi c.337+1G>A | 12 |
| 181 | No | 209 | M | CGD | Defective DHR,  bimodal maternal DHR pattern | Sanger | *CYBB* | XL | Hemi c.271C>T, p.Arg91Ter | 12 |
| 182 | No | 210 | M | CGD | Defective DHR,  Bimodal maternal DHR pattern | Sanger | *CYBB* | XL | Hemi c.359T>C, p.Leu120pro | 12 |
| 183 | No | 211 | M | CGD | Defective DHR,  Bimodal maternal DHR pattern | Sanger | *CYBB* | XL | Hemi c.1139G>A, p.Trp380Ter | 12 |
| 184 | No | 212 | M | CGD | Defective DHR,  Bimodal maternal DHR pattern | Sanger | *CYBB* | XL | Hemi Deletion exon 2 | 12 |
| 185 | No | 213 | M | CGD | Defective DHR,  Bimodal maternal DHR pattern | Sanger | *CYBB* | XL | Hemi c.1598_1600delGAG, p.Gly533del | 12 |
|  |  | 214 | M | CGD | Defective DHR,  Bimodal maternal DHR pattern | Sanger | *CYBB* | XL | Hemi c.1598_1600delGAG, p.Gly533del |  |
| 186 | Yes | 215 | F | CGD | Defective DHR, Defective CYBA | WES | *CYBA* | AR | Homo c.160_161InsC, p.Tyr54SerfsTer159 | 12 |
| 187 | Yes | 216 | M | CGD | Defective DHR, Defective CYBA | Sanger | *CYBA* | AR | Homo c.295_301delGTGCCCG, p.Val99ProfsTer90 | 12 |
| 188 | Yes | 217 | F | CGD | Defective DHR, Defective CYBA | Sanger | *CYBA* | AR | Homo c.295_301delGTGCCCG, p.Val99ProfsTer90 | 12 |
| 189 | Yes | 218 | F | CGD | Defective DHR, Defective CYBA | Sanger | *CYBA* | AR | Homo c.295_301delGTGCCCG, p.Val99ProfsTer90 | 12 |
| 190 | Yes | 219 | M | CGD | Defective DHR, Defective CYBA | Sanger | *CYBA* | AR | Homo c.295_301delGTGCCCG, p.Val99ProfsTer90 | 12 |
| 191 | Yes | 220^a^ | F | CGD | Defective DHR, Defective CYBA | Sanger | *CYBA* | AR | Homo c.295_301delGTGCCCG, p.Val99ProfsTer90 | 12 |
|  |  | 221^b^ | F | CGD | Defective DHR, Defective CYBA | Sanger | *CYBA* | AR | Homo c.295_301delGTGCCCG, p.Val99ProfsTer90 | 12 |
| 192 | Yes | 222^a^ | F | CGD | Defective DHR, Defective CYBA | Sanger | *CYBA* | AR | Homo c.295_301delGTGCCCG, p.Val99ProfsTer90 | 12 |
|  |  | 223^b^ | F | CGD | Defective DHR, Defective CYBA | Sanger | *CYBA* | AR | Homo c.295_301delGTGCCCG, p.Val99ProfsTer90 | 12 |
| 193 | Yes | 224 | M | CGD | Defective DHR, Defective CYBA | Sanger | *CYBA* | AR | Homo c.295_301delGTGCCCG, p.Val99ProfsTer90 | 12 |
| 194 | Yes | 225 | M | CGD | Defective DHR, Defective CYBA | Sanger | *CYBA* | AR | Homo c.295_301delGTGCCCG, p.Val99ProfsTer90 | 12 |
| 195 | Yes | 226 | M | CGD | Defective DHR, Defective CYBA | Sanger | *CYBA* | AR | Homo c.295_301delGTGCCCG, p.Val99ProfsTer90 |  |
| 196 | Yes | 227 | F | CGD | Defective DHR, Defective CYBA | Sanger | *CYBA* | AR | Homo c.295_301delGTGCCCG, p.Val99ProfsTer90 |  |
| 197 | Yes | 228 | M | CGD | Defective DHR, Defective CYBA | Sanger | *CYBA* | AR | Homo c.295_301delGTGCCCG, p.Val99ProfsTer90 |  |
| 198 | Yes | 229^a^ | F | CGD | Defective DHR, Defective CYBA | Sanger | *CYBA* | AR | Homo c.295_301delGTGCCCG, p.Val99ProfsTer90 |  |
|  |  | 230^b^ | F | CGD | Defective DHR, Defective CYBA | Sanger | *CYBA* | AR | Homo c.295_301delGTGCCCG, p.Val99ProfsTer90 |  |
| 199 | Yes | 231 | M | CGD | Defective DHR, Defective CYBA | Sanger | *CYBA* | AR | Homo c.295_301delGTGCCCG, p.Val99ProfsTer90 |  |
| 200 | Yes | 232 | F | CGD | Defective DHR, Defective CYBA | Sanger | *CYBA* | AR | Homo c.295_301delGTGCCCG, p.Val99ProfsTer90 |  |
| 201 | No | 233 | F | CGD | Defective DHR, Defective CYBA | Sanger | *CYBA* | AR | Homo c.295_301delGTGCCCG, p.Val99ProfsTer90 |  |
| 202 | Yes | 234 | F | CGD | Defective DHR, Defective CYBA | Sanger | *CYBA* | AR | Homo c.295_301delGTGCCCG, p.Val99ProfsTer90 |  |
| 203 | No | 235 | F | CGD | Defective DHR, Defective CYBA | Sanger | *CYBA* | AR | Homo c.295_301delGTGCCCG, p.Val99ProfsTer90 |  |
| 204 | Yes | 236 | F | CGD | Defective DHR, Defective CYBA | Sanger | *CYBA* | AR | Homo c.383-393delCACTGCTCGCC, p.Gly128AspfsTer81 | 12 |
| 205 | Yes | 237 | F | CGD | Defective DHR, Defective NCF1 | Sanger | *NCF1* | AR | Homo c.75_76delGT,p.Tyr26HisfsTer26 | 12 |
| 206 | Yes | 238^a^ | F | CGD | Defective DHR, Defective NCF1 | Sanger | *NCF1* | AR | Homo c.75_76delGT,p.Tyr26HisfsTer26 | 12 |
|  |  | 239^b^ | M | CGD | Defective DHR, Defective NCF1 | Sanger | *NCF1* | AR | Homo c.75_76delGT,p.Tyr26HisfsTer26 | 12 |
| 207 | Yes | 240^a^ | M | CGD | Defective DHR, Defective NCF1 | Sanger | *NCF1* | AR | Homo c.75_76delGT,p.Tyr26HisfsTer26 | 12 |
|  |  | 241^b^ | F | CGD | Defective DHR, Defective NCF1 | Sanger | *NCF1* | AR | Homo c.75_76delGT,p.Tyr26HisfsTer26 | 12 |
| 208 | Yes | 242 | F | CGD | Defective DHR, Defective NCF1 | Sanger | *NCF1* | AR | Homo c.75_76delGT,p.Tyr26HisfsTer26 | 12 |
| 219 | Yes | 243 | M | CGD | Defective DHR, Defective NCF1 | Sanger | *NCF1* | AR | Homo c.75_76delGT,p.Tyr26HisfsTer26 | 12 |
| 210 | Yes | 244^a^ | M | CGD | Defective DHR, Defective NCF1 | Sanger | *NCF1* | AR | Homo c.75_76delGT,p.Tyr26HisfsTer26 | 12 |
|  |  | 245^b^ | M | CGD | Defective DHR, Defective NCF1 | Sanger | *NCF1* | AR | Homo c.75_76delGT,p.Tyr26HisfsTer26 |  |
| 211 | Yes | 246 | F | CGD | Defective DHR, Defective NCF1 | Sanger | *NCF1* | AR | Homo c.75_76delGT,p.Tyr26HisfsTer26 | 12 |
| 212 | No | 247^a^ | F | CGD | Defective DHR, Defective NCF1 | Sanger | *NCF1* | AR | Homo c.75_76delGT,p.Tyr26HisfsTer26 | 12 |
|  |  | 248^b^ | M | CGD | Defective DHR, Defective NCF1 | Sanger | *NCF1* | AR | Homo c.75_76delGT,p.Tyr26HisfsTer26 | 12 |
| 213 | Yes | 259 | M | CGD | Defective DHR, Defective NCF1 | Sanger | *NCF1* | AR | Homo c.75_76delGT,p.Tyr26HisfsTer26 |  |
| 214 | Yes | 250 | F | CGD | Defective DHR, Defective NCF2 | Sanger | *NCF2* | AR | Homo c.239T>C, p.Leu80Pro | 12 |
| 215 | Yes | 251 | M | CGD | Defective DHR, Defective NCF2 | Sanger | *NCF2* | AR | Homo c.574C>T, p.Gln192Ter | 12 |
| 216 | Yes | 252 | M | LAD | Defective CD11b/CD18 | Sanger | *ITGB2* | AR | Homo c.1377C>A, p.Cys459Ter |  |
| 217 | Yes | 253 | F | LAD | Defective CD11b/CD18 | Sanger | *ITGB2* | AR | Homo c.652C>T, p.Gln218Ter |  |
| 218 | Yes | 254 | F | LAD | Defective CD11b/CD18 | Sanger | *ITGB2* | AR | Homo c.500G>T, p.Gly167Val |  |
| 229 | Yes | 255 | M | LAD | Defective CD11b/CD18 | Sanger | *ITGB2* | AR | Homo c.505G>A, p.Gly169Arg |  |
| 220 | Yes | 256 | F | LAD | Defective CD11b/CD18 | Sanger | *ITGB2* | AR | Homo c.505G>A, p.Gly169Arg |  |
| 221 | Yes | 257 | F | LAD | Defective CD11b/CD18 | Sanger | *ITGB2* | AR | Homo c.505G>A, p.Gly169Arg |  |
| 222 | No | 258 | M | LAD | Defective CD11b/CD18 | Sanger | *ITGB2* | AR | Homo c.307delG, p.Val103Ter |  |
| 223 | Yes | 259 | F | LAD | Defective CD11b/CD18 | Sanger | *ITGB2* | AR | Homo c.306dup, p.Val103SerfsTer39 |  |
| 224 | Yes | 260 | F | LAD | Defective CD11b/CD18 | Sanger | *ITGB2* | AR | Homo c.185G>A, p.Cys62Tyr |  |
| 225 | Yes | 261 | M | LAD | Defective CD11b/CD18 | Sanger | *ITGB2* | AR | Homo c.185G>A, p.Cys62Tyr |  |
| Defects in intrinsic and innate immunity | | | | | | | | | | |
| 226 | No | 262 | M | CMC | Normal immunophenotype &DHR | Sanger | *STAT1* | AD GOF | Hetero c.862A>C, p.Thr288Pro |  |
| 227 | No | 263 | F | CMC | Normal immunophenotype &DHR | Sanger | *STAT1* | AD GOF | Hetero c.1154C>T, p.Thr385Met |  |
| 228 | Yes | 264 | M | CMC | Normal immunophenotype &DHR | Sanger | *STAT1* | AD GOF | Hetero c.1198C>G, p.Leu400Val |  |
| 229 | Yes | 265 | M | CMC | Normal immunophenotype &DHR | Sanger | *STAT1* | AD GOF | Hetero c.1199T>A, p.Leu400Gln |  |
| 230 | Yes | 266 | F | CID | Normal immunophenotype &DHR | WES | *STAT2* | AR | Homo c.512A>T, p.Asp171Val |  |
| 231 | Yes | 267^a^ | F | MSMD | Normal immunophenotype &DHR | WES | *IL12RB1* | AR | Homo c.64+2T>G |  |
|  |  | 268^b^ | M | MSMD | Normal immunophenotype &DHR | WES | *IL12RB1* | AR | Homo c.64+2T>G |  |
| 232 | Yes | 269 | M | MSMD | Normal immunophenotype &DHR | WES | *IL12RB1* | AR | Homo c.64+2T>G |  |
| 233 | Yes | 270 | M | MSMD | Normal immunophenotype &DHR | WES | *IL12RB1* | AR | Homo c.64+2T>G |  |
| 234 | Yes | 271 | M | MSMD | Normal immunophenotype &DHR | WES | *IL12RB1* | AR | Homo c.643C>T, p.Arg215Trp |  |
| 235 | Yes | 272 | M | ALPS like | Increase Double negative αβ T cells | WES | *IL12RB1* | AR | Homo c.1791+2T>G |  |
| 236 | Yes | 273 | M | MSMD | Normal immunophenotype &DHR | WES | *IFNGR2* | AR | Homo c.371C>T, p.Ser124Phe |  |
| 237 | Yes | 274 | F | MSMD | Normal immunophenotype &DHR | WES | *IFNGR1* | AR | NA |  |
| Autoinflammatory disorders | | | | | | | | | | |
| 238 | No | 275 | M | Immune dysregulation | Normal immunophenotype, | NGS | *STING1* | AD | Hetero c.575G>T, p.Gly192Val |  |
| 239 | No | 276 | M | Immune dysregulation | Normal immunophenotype,  Low Tregs | NGS | *PLCG2* | AD | Hetero c.886delT, p.Ser296HisfrTer19 |  |
|  |  |  |  |  |  |  | *HUWE1* | XL | Hemi c.12913A>G, p.Lys4305Glu |  |
| 240 | No | 277 | M | Autoinflammatory disorder | Normal immunophenotype,  Normal Tregs | NGS | *NLRP3* | AD | Hetero c.584C>T, p.Thr195Met |  |
| 241 | Yes | 278 | F | Immune dysregulation | Normal immunophenotype | NGS | *NLRP12* | AD | Hetero c.209G>A, p.Trp70Ter |  |
| 242 | Yes | 279 | M | CID | CD4 lymphopenia | NGS | *PSTPIP1* | AD | Hetero c.59C>T, p.Thr20Met |  |
| 243 | No | 280 | M | CID/Immune dysregulation | Normal immunophenotype,  Normal DOCK8 | NGS | *SH3BP2* | AD | Hetero c.1103delC, p.Pro368GlnfsTer21 |  |
| Complement deficiencies | | | | | | | | | | |
| 244 | Yes | 281 | F | CID | CD4 lymphopenia | WES | *CD59* | AR | Homo c.80delA, p.Gln27ArgfsTer53 |  |
| Others | | | | | | | | | | |
| 245 | Yes | 282 | F | SCID | T-B+NK+ | WES | *THEMIS* | AR | Homo c.1004C>A, p.Pro335His |  |
| Patients with genetic diagnosis not reached | | | | | | | | | | |
| 1 | Yes | 1 | M | Omenn | Low CD19 | Sanger | *RAG1/2* | AR | *RAG2* Hetero c.909G>T, p.Glu303Asp |  |
| 2 | Yes | 2 | F | Omenn/SCID | T-B-NK- | Sanger | *RAG1/2* | AR | No pathogenic variant |  |
| 3 | Yes | 3 | F | SCID | T-B-NK+ | Sanger | *RAG1/2* | AR | No pathogenic variant |  |
| 4 | Yes | 4 | M | SCID | T-B-NK+ | Sanger | *RAG1/2* | AR | No pathogenic variant |  |
| 5 | Yes | 5 | M | SCID | T-B+NK+ | Sanger | *RAG1/2* | AR | No pathogenic variant |  |
|  |  |  |  |  |  |  | *IL7RA* | AR | No pathogenic variant |  |
| 6 | Yes | 6 | F | SCID | T-B+NK+ | Sanger | *RAG1/2* | AR | No pathogenic variant |  |
| 7 | Yes | 7 | F | SCID | T-B-NK+ | Sanger | *RAG1/2* | AR | No pathogenic variant |  |
| 8 | Yes | 8 | M | SCID | T-B-NK+ | Sanger | *RAG1/2* | AR | No pathogenic variant |  |
| 9 | No | 9 | F | SCID | T-B+ | Sanger | *RAG1/2* | AR | No pathogenic variant |  |
|  |  |  |  |  |  |  | *IL7RA* | AR | No pathogenic variant |  |
| 10 | Yes | 10 | M | SCID | T-B-NK+ | Sanger | *RAG1/2* | AR | No pathogenic variant |  |
| 11 | Yes | 11 | F | SCID | T-B-NK+ | Sanger | *RAG1/2* | AR | No pathogenic variant |  |
| 12 | Yes | 12 | F | SCID | T-B-NK+ | Sanger | *RAG1/2* | AR | No pathogenic variant |  |
| 13 | No | 13 | M | SCID | T-B-NK+ | Sanger | *RAG1/2* | AR | No pathogenic variant |  |
| 14 | Yes | 14 | M | SCID | T-B-NK+ | Sanger | *RAG1/2* | AR | No pathogenic variant |  |
| 15 | Yes | 15 | M | SCID | T-B-NK+ | Sanger | *RAG1/2* | AR | No pathogenic variant |  |
|  |  |  |  |  |  |  | *DCLRE1C* | AR | No pathogenic variant |  |
| 16 | Yes | 16 | M | SCID | T-B- | Sanger | *RAG1/2* | AR | No pathogenic variant |  |
|  |  |  |  |  |  |  | *ADA* | AR | No pathogenic variant |  |
| 17 | Yes | 17 | F | SCID | T-B- | Sanger | *RAG1/2* | AR | No pathogenic variant |  |
|  |  |  |  |  |  |  | *ADA* | AR | No pathogenic variant |  |
| 18 | Yes | 18 | M | SCID | T-B-NK- | Sanger | *PNP* | AR | No pathogenic variant |  |
|  |  |  |  |  |  |  | *ADA* | AR | No pathogenic variant |  |
| 19 | Yes | 19 | M | SCID | T-B-NK- | Sanger | *ADA* | AR | No pathogenic variant |  |
| 20 | Yes | 20 | M | SCID | T-B-NK- | Sanger | *ADA* | AR | No pathogenic variant |  |
| 21 | Yes | 21 | M | SCID | T-B-NK | Sanger | *ADA* | AR | Hetero c.58G>A, p.Gly20Arg |  |
| 22 | Yes | 22 | M | SCID | T-B+NK- | Sanger | *JAK3* | AR | No pathogenic variant |  |
| 23 | Yes | 23 | F | SCID | T-B+ | WES | *MEFV* | AR | Hetero c.2082C>T, p.Met694Ile |  |
| 24 | No | 24 | F | SCID | T-B+NK+ | WES |  |  | No pathogenic variant |  |
| 25 | No | 25 | M | SCID | T-B+NK- | WES |  |  | No pathogenic variant |  |
| 26 | Yes | 26^a^ | F | SCID | T-B+NK- | WES | *JAK3* | AR | Hetero c.2141C>T, p.Thr714Met |  |
|  |  | 27^b^ | M | SCID | T-B+NK- | WES |  |  | No pathogenic variant |  |
| 27 | Yes | 28 | F | SCID | T-B+NK+ | WES |  |  | No pathogenic variant |  |
| 28 | No | 29 | F | SCID | T-B+NK+ | WES | *JAK3* | AR | Hetero c.564G>C, p.Val188Val |  |
| 29 | No | 30 | F | SCID | T-B+NK+ | WES |  |  | No pathogenic variant |  |
| 30 | Yes | 31 | M | SCID | T-B+NK- | WES |  |  | No pathogenic variant |  |
| 31 | Yes | 32 | F | SCID | T-B+NK- | NGS |  |  | No pathogenic variant |  |
| 32 | No | 33 | M | SCID | T-B+NK+ | Sanger | *IL7RA* | AR | No pathogenic variant |  |
| 33 | Yes | 34 | M | SCID | T-B+NK+ | Sanger | *IL7RA* | AR | No pathogenic variant |  |
| 34 | Yes | 35 | M | SCID | T-B+NK+ | Sanger | *IL7RA* | AR | No pathogenic variant |  |
| 35 | Yes | 36 | F | SCID | T-B+NK+ | Sanger | *IL7RA* | AR | No pathogenic variant |  |
| 36 | Yes | 37 | M | SCID | T-B+NK+ | Sanger | *IL7RA* | AR | No pathogenic variant |  |
| 37 | Yes | 38 | F | SCID | T-B+NK- | Sanger | *IL7RA* | AR | No pathogenic variant |  |
| 38 | Yes | 39 | M | SCID | T-B+NK+ | Sanger | *IL7RA* | AR | No pathogenic variant |  |
| 39 | Yes | 40 | M | SCID | T-B+ | Sanger | *IL7RA* | AR | No pathogenic variant |  |
|  |  |  |  |  |  | WES |  |  | No pathogenic variant |  |
| 40 | Yes | 41 | F | SCID | T-B+NK+ | Sanger | *IL7RA* | AR | No pathogenic variant |  |
|  |  |  |  |  |  | WES |  |  | No pathogenic variant |  |
| 41 | Yes | 42 | F | SCID | T-B+NK+ | WES |  |  | No pathogenic variant |  |
| 42 | No | 43 | M | SCID | T-B+NK+ | Sanger | *IL7RA* | AR | No pathogenic variant |  |
|  |  |  |  |  |  | WES |  |  | No pathogenic variant |  |
| 43 | Yes | 44 | M | SCID | T-B+NK+ | WES |  |  | No pathogenic variant |  |
| 44 | Yes | 45 | F | SCID | T-B+NK+ | WES |  |  | No pathogenic variant |  |
| 45 | Yes | 46 | F | SCID | T-B-NK- | WES |  |  | No pathogenic variant |  |
| 46 | Yes | 47 | F | SCID | T-B+NK- | WES |  |  | No pathogenic variant |  |
| 47 | Yes | 48 | F | SCID | Low RTE, Normal DHR | WES |  |  | No pathogenic variant |  |
| 48 | Yes | 49 | F | SCID/ 2 siblings death (SCID) | Low RTE | WES |  |  | No pathogenic variant |  |
| 49 | Yes | 50 | F | SCID/CID | T-B-NK- | Sanger | *PNP* | AR | No pathogenic variant |  |
|  |  |  |  |  |  |  | *ADA* | AR | No pathogenic variant |  |
| 50 | Yes | 51 | M | SCID/CID | Absolute lymphopenia, T-B-NK+ | Sanger | *RAG1/2* | AR | No pathogenic variant |  |
| 51 | No | 52 | F | SCID/CID | T-B+ | Sanger | *IL7RA* | AR | No pathogenic variant |  |
|  |  |  |  |  |  | WES |  |  | No pathogenic variant |  |
| 52 | Yes | 53 | F | SCID/CID | T-B+NK+ | Sanger | *IL7RA* | AR | No pathogenic variant |  |
| 53 | Yes | 54 | M | SCID/CID | CD4 lymphopenia, Low CD19 | Sanger | *RAG1/2* | AR | No pathogenic variant |  |
| 54 | No | 55 | M | SCID/CID | CD4 lymphopenia | Sanger | *RAG1/2* | AR | No pathogenic variant |  |
| 55 | Yes | 56 | M | SCID/CID | CD4 lymphopenia | NGS |  |  | No pathogenic variant |  |
| 56 | Yes | 57 | M | Atypical SCID/  CID | Absolute lymphopenia,  CD4 lymphopenia, Low CD19 | Sanger | *RAG1/2* | AR | No pathogenic variant |  |
| 57 | No | 58 | M | Atypical SCID/  CID | CD4 lymphopenia, Low CD19 | Sanger | *RAG1/2* | AR | No pathogenic variant |  |
|  |  |  |  |  |  | FISH |  |  | Mixed chimerism |  |
| 58 | Yes | 59 | M | Atypical SCID/  CID | Absolute lymphopenia | Sanger | *RAG1/2* | AR | No pathogenic variant |  |
|  |  |  |  |  |  |  | *ADA* | AR | No pathogenic variant |  |
| 59 | Yes | 60 | M | Atypical SCID/  CID | CD4 lymphopenia, Normal HLADR, Normal DHR | Sanger | *RAG1/2* | AR | No pathogenic variant |  |
| 60 | Yes | 61 | F | CID | CD4 Lymphopenia | WES |  |  | No pathogenic variant |  |
| 61 | Yes | 62 | M | CID | CD4 Lymphopenia | Sanger | *PNP* | AR | No pathogenic variant |  |
|  |  |  |  |  |  |  | *ADA* | AR | No pathogenic variant |  |
|  |  |  |  |  |  | WES | *TCF3* | AD | Hetero c.625A>G, p.Thr209Ala (under analysis) |  |
| 62 | Yes | 63 | F | CID | Absolute lymphopenia | WES |  |  | No pathogenic variant |  |
| 63 | No | 64^a^ | M | CID | Normal immunophenotyping, Normal DHR, Normal DOCK8 | WES |  |  | No pathogenic variant |  |
|  |  | 65^b^ | M | CID | Normal immunophenotyping, Normal DHR, Normal DOCK8 | WES |  |  | No pathogenic variant |  |
| 64 | No | 66 | M | CID | Low T cells/  Increase αβ double negative T cells | WES |  |  | No pathogenic variant |  |
| 65 | Yes | 67 | F | CID | CD4 lymphopenia, Normal DHR | Sanger | *STAT1* |  | No pathogenic variant |  |
| 66 | Yes | 68 | F | CID | CD4 lymphopenia | NGS |  |  |  |  |
| 67 | Yes | 69 | F | CID | Low CD3 | NGS |  |  | No pathogenic variant |  |
| 68 | Yes | 70 | M | CID | CD4 lymphopenia, Low CD19 | Sanger | *RAG1/2* | AR | No pathogenic variant |  |
|  |  |  |  |  |  |  | *PNP* | AR | No pathogenic variant |  |
|  |  |  |  |  |  | NGS |  |  | No pathogenic variant |  |
| 69 | Yes | 71 | M | CID | CD4 lymphopenia, Low CD19 | Sanger | *RAG1/2* | AR | No pathogenic variant |  |
| 70 | Yes | 72 | M | CID | CD4 lymphopenia, Low CD19 | Sanger | *RAG1/2* | AR | No pathogenic variant |  |
| 71 | Yes | 73 | F | CID | CD4 lymphopenia, Low CD19 | Sanger | *RAG1/2* | AR | No pathogenic variant |  |
| 72 | Yes | 74 | M | CID | CD4 lymphopenia, Low CD19 | Sanger | *RAG1/2* | AR | No pathogenic variant |  |
| 73 | Yes | 75 | F | CID | CD4 lymphopenia, Low CD19 | Sanger | *RAG1/2* | AR | No pathogenic variant |  |
| 74 | No | 76 | F | CID | Low CD19, Normal DHR | Sanger | *RAG1/2* | AR | No pathogenic variant |  |
| 75 | Yes | 77 | M | CID | CD4 lymphopenia, Low CD19 | Sanger | *RAG1/2* | AR | No pathogenic variant |  |
| 76 | Yes | 78 | F | CID | Low CD19 | Sanger | *RAG1/2* | AR | No pathogenic variant |  |
| 77 | No | 79 | F | CID | Low CD19 | Sanger | *RAG1/2* | AR | No pathogenic variant |  |
| 78 | Yes | 80 | M | CID | CD4 lymphopenia, Low CD19 | Sanger | *RAG1/2* | AR | No pathogenic variant |  |
|  |  |  |  |  |  |  | *ADA* | AR | Hetero c.22G>A, p.Asp8Asn |  |
| 79 | Yes | 81 | M | CID | Low CD19 | Sanger | *RAG1/2* | AR | No pathogenic variant |  |
|  |  |  |  |  |  | NGS |  |  | No pathogenic variant |  |
| 80 | Yes | 82 | F | CID | CD4 lymphopenia | Sanger | *PNP* | AR | No pathogenic variant |  |
|  |  |  |  |  |  |  | *ADA* | AR | No pathogenic variant |  |
| 81 | Yes | 83 | F | CID | Low CD3 | Sanger | *PNP* | AR | No pathogenic variant |  |
|  |  |  |  |  |  |  | *ADA* | AR | No pathogenic variant |  |
| 82 | Yes | 84 | M | CID | CD4 lymphopenia | Sanger | *PNP* | AR | No pathogenic variant |  |
|  |  |  |  |  |  |  | *ADA* | AR | No pathogenic variant |  |
| 83 | Yes | 85 | F | CID | T-B-NK+ | Sanger | *PNP* | AR | No pathogenic variant |  |
|  |  |  |  |  |  |  | *ADA* | AR | No pathogenic variant |  |
| 84 | Yes | 86 | F | CID | CD4 lymphopenia, Low CD19 | NGS | *ADA* | AR | Hetero c.466C>T, p.Arg156Cys |  |
| 85 | Yes | 87 | F | CID | CD4 lymphopenia, Normal DOCK8, Normal DHR | NGS |  |  | No pathogenic variant |  |
| 86 | No | 88 | F | CID | CD4 lymphopenia | NGS |  |  | No pathogenic variant |  |
| 87 | Yes | 89 | M | CID | CD4 lymphopenia | NGS |  |  | No pathogenic variant |  |
| 88 | Yes | 90 | M | CID | CD4 lymphopenia, Normal DHR | NGS |  |  | No pathogenic variant |  |
| 89 | Yes | 91 | M | CID | CD4 lymphopenia, Low CD19 | NGS |  |  | No pathogenic variant |  |
| 90 | Yes | 92 | M | CID | CD4 lymphopenia | NGS |  |  | No pathogenic variant |  |
| 91 | Yes | 93 | M | CID | CD4 lymphopenia | NGS |  |  | No pathogenic variant |  |
| 92 | Yes | 94 | F | CID | CD4 lymphopenia | WES |  |  | No pathogenic variant |  |
| 93 | Yes | 95 | F | CID | Normal immunophenotyping, DHR | WES | *LAMA3* | AR | Hetero H280D, hetero L269Del |  |
| 94 | No | 96 | M | CID/HIES | Normal immunophenotyping, DHR | WES |  |  | No pathogenic variant |  |
| 95 | Yes | 97 | M | CID/HIES | CD4 lymphopenia, Normal DOCK8, Normal DHR | NGS |  |  | No pathogenic variant |  |
|  |  |  |  |  |  | WES |  |  | No pathogenic variant |  |
| 96 | Yes | 98 | F | CID/HIES | CD4 lymphopenia | NGS |  |  | No pathogenic variant |  |
| 97 | Yes | 99 | M | CID/HIES | Normal immunophenotype | NGS |  |  | No pathogenic variant |  |
| 98 | Yes | 100^a^ | M | CID/HIES | Normal immunophenotype | NGS |  |  | No pathogenic variant |  |
|  |  | 101^b^ | M | CID/HIES | Normal immunophenotype | NGS |  |  | No pathogenic variant |  |
| 99 | Yes | 102 | M | CID/HIES | CD4 lymphopenia,  Defective DOCK8 | NGS |  |  | No pathogenic variant |  |
| 100 | Yes | 103 | F | CID/HIES | CD4 lymphopenia,  Defective DOCK8 | NGS |  |  | No pathogenic variant |  |
| 101 | Yes | 104 | M | CID/HIES | CD4 lymphopenia,  Defective DOCK8 | NGS |  |  | No pathogenic variant |  |
| 102 | Yes | 105 | F | CID/HIES | CD4 lymphopenia | NGS |  |  | No pathogenic variant |  |
|  | Yes | 106 | F | CID/HIES | CD4 lymphopenia | NGS |  |  | No pathogenic variant |  |
| 103 | Yes | 107^a^ | M | CID/HIES | Normal immunophenotype | NGS |  |  | No pathogenic variant |  |
|  |  | 108^b^ | F | CID/HIES | Normal immunophenotype | NGS |  |  | No pathogenic variant |  |
|  |  | 109^c^ | M | CID/HIES | Normal immunophenotype | NGS |  |  | No pathogenic variant |  |
| 104 | Yes | 110 | F | CID with syndromic feature | Normal immunophenotype | Sanger | *PNP* | AR | No pathogenic variant |  |
|  |  |  |  |  |  | WES | *SURF1* | AR | Homo variant |  |
| 105 | No | 111 | M | DiGeorge syndrome | Low CD3 | FISH | Chr. 22q11 |  | Normal |  |
| 106 | Yes | 112 | M | DiGeorge syndrome | Normal immunophenotype | FISH | Chr. 22q11 |  | Normal |  |
| 107 | Yes | 113 | F | DiGeorge/  Charge syndrome | CD4 lymphopenia | FISH | Chr. 22q11 |  | Normal |  |
| 108 | Yes | 114 | M | HIGM | Normal immunophenotype | Sanger | *RAG1/2* | AR | No pathogenic variant |  |
|  |  |  |  |  |  | NGS |  |  | No pathogenic variant |  |
| 109 | Yes | 115 | M | HIGM | Normal immunophenotype | NGS |  |  | No pathogenic variant |  |
| 110 | Yes | 116 | M | CID/ Immune dysregulation | CD4 lymphopenia, Normal LRBA | Sanger | *RAG1/2* | AR | No pathogenic variant |  |
|  |  |  |  |  |  | NGS |  |  | No pathogenic variant |  |
| 111 | No | 117 | M | CID/immune dysregulation | Normal immunophenotype, Low Tregs | WES |  |  | No pathogenic variant |  |
| 112 | No | 118^a^ | F | CID/immune dysregulation | CD4 Lymphopenia, Normal LRBA | WES |  |  | No pathogenic variant |  |
|  |  | 119^b^ | F | CID/Immune dysregulation | CD4 Lymphopenia, Normal LRBA | WES |  |  | No pathogenic variant |  |
| 113 | Yes | 120 | M | XLA | Low CD19, Normal BTK | NGS |  |  | No pathogenic variant |  |
| 114 | Yes | 121^a^ | M | XLA | Low CD19, Normal BTK | NGS |  |  | No pathogenic variant |  |
|  |  | 122^b^ | M | XLA | Low CD19, Normal BTK | NGS |  |  | No pathogenic variant |  |
| 115 | Yes | 123 | F | Antibody deficiency | Low CD19 | NGS |  |  | No pathogenic variant |  |
| 116 | No | 124 | M | Antibody deficiency | CD4 lymphopenia | NGS |  |  | No pathogenic variant |  |
| 117 | Yes | 125 | M | Antibody deficiency | Normal immunophenotype | NGS |  |  | No pathogenic variant |  |
| 118 | No | 126 | F | Antibody deficiency | Normal immunophenotype | NGS |  |  | No pathogenic variant |  |
| 119 | Yes | 127 | M | Antibody deficiency | Low CD19 | Sanger | *RAG1/2* | AR | No pathogenic variant |  |
| 120 | Yes | 128 | F | CVID | Absolute lymphopenia, Low CD19 | NGS |  |  | No pathogenic variant |  |
| 121 | Yes | 129 | F | CVID | Normal Immunophenotype,  Normal LRBA | NGS |  |  | No pathogenic variant |  |
| 122 | Yes | 130 | F | IBD | Normal immunophenotype,  Normal DHR | Sanger | *IL10RA/RB* | AR | No pathogenic variant |  |
|  |  |  |  |  |  | NGS |  |  | No pathogenic variant |  |
| 123 | No | 131 | M | IBD | Normal immunophenotype | NGS |  |  | No pathogenic variant |  |
| 124 | Yes | 132 | F | EO-IBD | Normal immunophenotype,  Normal LRBA, Normal DHR | Sanger | *IL10RA/RB* | AR | No pathogenic variant |  |
| 125 | No | 133 | M | EO-IBD | Normal immunophenotype,  Normal DHR | Sanger | *IL10RA/RB* | AR | No pathogenic variant |  |
| 126 | No | 134 | F | EO-IBD | Normal immunophenotype,  Normal DHR | Sanger | *IL10RA/RB* | AR | No pathogenic variant |  |
| 127 | No | 135 | M | EO-IBD | Normal immunophenotype,  Normal DHR | Sanger | *IL10RA/RB* | AR | No pathogenic variant |  |
| 128 | Yes | 136 | F | EO-IBD | Normal immunophenotype,  Normal DHR | Sanger | *IL10RA/RB* | AR | No pathogenic variant |  |
| 129 | Yes | 137 | F | EO-IBD | Normal immunophenotype,  Normal DHR | Sanger | *IL10RA/RB* | AR | No pathogenic variant |  |
| 130 | Yes | 138 | F | EO-IBD | Normal immunophenotype,  Normal DHR, Low Tregs | NGS |  |  | No pathogenic variant |  |
| 131 | Yes | 139 | F | VEO-IBD | Normal immunophenotype | Sanger | *IL10RA/RB* | AR | No pathogenic variant |  |
|  |  |  |  |  |  | NGS |  |  | No pathogenic variant |  |
| 132 | No | 140 | M | VEO-IBD | Normal immunophenotype, Defective Tregs, Normal DHR | NGS |  |  | No pathogenic variant |  |
| 133 | Yes | 141 | M | Immune dysregulation | Normal immunophenotype,  Normal DHR | Sanger | *IL10RA/RB* | AR | No pathogenic variant |  |
| 134 | Yes | 142 | F | Immune dysregulation | Normal immunophenotype,  Normal DHR | Sanger | *IL10RA/RB* | AR | No pathogenic variant |  |
| 135 | Yes | 143 | F | Immune dysregulation | Normal immunophenotype | WES |  |  | No pathogenic variant |  |
| 136 | Yes | 144 | F | Immune dysregulation | CD4 lymphopenia,  Normal LRBA, Normal DOCK8 | NGS | *MEFV* | AR | Heterozygous c.2082G>A, p.Met694Ile |  |
|  |  |  |  |  |  |  | *DOCK8* | AR | Hetero c.626G>A, p.Arg209Gln |  |
| 137 | No | 145 | F | Immune dysregulation | Low CD19, Normal LRBA | NGS |  |  | No pathogenic variant |  |
| 138 | Yes | 146 | F | Immune dysregulation | Low CD19, Normal LRBA,  Normal DOCK8, Normal DHR | NGS |  |  | No pathogenic variant |  |
| 139 | No | 147 | M | Immune dysregulation | Normal immunophenotype,  Low Tregs | NGS |  |  | No pathogenic variant |  |
| 140 | Yes | 148 | M | Immune dysregulation | Normal immunophenotype,  Normal LRBA | Sanger | *PNP* | AR | No pathogenic variant |  |
|  |  |  |  |  |  | NGS |  |  | No pathogenic variant |  |
| 141 | No | 149 | M | Immune dysregulation | Normal immunophenotype,  Normal LRBA | NGS |  |  | No pathogenic variant |  |
| 142 | No | 150 | M | Immune dysregulation | Normal immunophenotype,  Normal LRBA | NGS | *MEFV* | AR | Heterozygous c.2230G>T, p.Ala744Ser |  |
| 143 | No | 151 | F | Immune dysregulation | Normal immunophenotype,  Normal DHR | NGS |  |  | No pathogenic variant |  |
| 144 | No | 152 | M | Immune dysregulation | Normal immunophenotype, DOCK8, Tregs | NGS |  |  | No pathogenic variant |  |
| 145 | No | 153 | M | Immune dysregulation | Normal immunophenotype, absolute lymphopenia, Low Tregs | NGS |  |  | No pathogenic variant |  |
| 146 | Yes | 154^a^ | M | Immune dysregulation | CD4 lymphopenia | NGS |  |  | No pathogenic variant |  |
|  |  | 155^b^ | M | Immune dysregulation | CD4 lymphopenia | NGS |  |  | No pathogenic variant |  |
| 147 | Yes | 156^a^ | F | Immune dysregulation | Normal immunophenotype | NGS |  |  | No pathogenic variant |  |
|  |  | 157^b^ | M | Immune dysregulation | Normal immunophenotype | NGS |  |  | No pathogenic variant |  |
| 148 | Yes | 158 | M | Immune dysregulation | Normal immunophenotype,  Normal DHR | NGS |  |  | No pathogenic variant |  |
| 149 | No | 159 | M | Immune dysregulation | CD4 lymphopenia, Normal LRBA | Sanger | *CTLA4* | AD | No pathogenic variant |  |
| 150 | Yes | 160 | F | Immune dysregulation | Normal immunophenotype | Sanger | *CTLA4* | AD | No pathogenic variant |  |
| 151 | No | 161 | F | Immune dysregulation | Low CD19, Normal LRBA | Sanger | *CTLA4* | AD | No pathogenic variant |  |
| 152 | No | 162 | F | Immune dysregulation | Low CD19 | Sanger | *CTLA4* | AD | No pathogenic variant |  |
| 153 | No | 163 | M | Immune dysregulation | Low CD19 | Sanger | *CTLA4* | AD | No pathogenic variant |  |
| 154 | Yes | 164 | F | Immune dysregulation | CD4 lymphopenia, Low CD19 | Sanger | *CTLA4* | AD | No pathogenic variant |  |
|  |  |  |  |  |  | NGS |  |  | No pathogenic variant |  |
| 155 | No | 165 | F | Immune dysregulation | Low CD19 | Sanger | *CTLA4* | AD | No pathogenic variant |  |
| 156 | Yes | 166 | M | Immune dysregulation | Normal immunophenotype | Sanger | *CTLA4* | AD | No pathogenic variant |  |
| 157 | No | 167 | M | CMC | Normal immunophenotype,  Normal DHR | WES |  |  | No pathogenic variant |  |
| 158 | No | 168 | F | CMC | Normal DHR | Sanger | *CARD9* | AR | No pathogenic variant |  |
|  |  |  |  |  |  | WES |  |  | Pending |  |
| 159 | Yes | 169 | F | CMC | Normal immunophenotype,  Normal DHR | WES |  |  |  |  |
| 160 | Yes | 170 | F | CMC | Normal immunophenotype,  Normal DHR | Sanger | *STAT1* |  | No pathogenic variant |  |
|  |  |  |  |  |  | WES | AIRE | AR | Hetero c.1099C>T, p.Pro367Ser |  |
| 161 | No | 171 | F | CMC | Normal immunophenotype,  Normal DHR | WES |  |  | No pathogenic variant |  |
| 162 | Yes | 172 | F | CMC | Normal immunophenotype | WES |  |  | No pathogenic variant |  |
| 163 | Yes | 173 | F | CMC | Normal DHR | WES |  |  | No pathogenic variant |  |
| 164 | No | 174^a^ | M | CMC | Normal immunophenotyping, DHR | Sanger | *CARD9* | AR | Hetero c.80C>T, Pro27Leu |  |
|  |  |  |  |  |  | WES | *GLA* | XL | Hemi c.647A>T, p.Tyr216Phe |  |
|  |  | 175^b^ | M | CMC | Normal immunophenotyping, DHR | Sanger | *CARD9* | AR | Hetero c.80C>T, Pro27Leu |  |
|  |  |  |  |  |  | WES | *GLA* | *XL* | Hemi c.647A>T, p.Tyr216Phe |  |
| 165 | No | 176 | M | CMC | Normal immunophenotyping | Sanger | *STAT1* |  | No pathogenic variant |  |
|  |  |  |  |  |  | WES |  |  | Pending |  |
| 166 | No | 177 | M | CMC | Normal DHR | Sanger | *CARD9* | AR | No pathogenic variant |  |
|  |  |  |  |  |  | WES |  |  | Pending |  |
| 167 | No | 178 | M | CMC | Normal immunophenotyping, DHR | WES |  |  | No pathogenic variant |  |
| 168 | No | 179 | M | CMC | Normal immunophenotyping, DHR | WES |  |  | No pathogenic variant |  |
| 169 | Yes | 180 | F | CMC | Normal immunophenotyping, DHR | WES |  |  | Under analysis |  |
| 170 | No | 181 | M | CMC | Normal DHR | Sanger | *STAT1* |  | No pathogenic variant |  |
|  |  |  |  |  |  |  | *CARD9* | AR | No pathogenic variant |  |
|  |  |  |  |  |  | WES |  |  | Under analysis |  |
| 171 | Yes | 182 | M | CMC | Normal immunophenotyping, DHR | WES |  |  | No pathogenic variant |  |
| 172 | Yes | 183 | M | CMC | Normal immunophenotyping, Normal DHR | WES |  |  | No pathogenic variant |  |
| 173 | Yes | 184 | M | CMC | CD4 lymphopenia, Low CD19, Normal DHR | WES | *OTULIN* | AR | Heterozygous variant |  |
| 174 | No | 185 | F | MSMD | Normal Immunophenotype | WES |  |  | No pathogenic variant |  |
| 175 | Yes | 186 | F | MSMD | Normal Immunophenotype | WES |  |  | No pathogenic variant |  |
| 176 | Yes | 187 | M | MSMD | Normal immunophenotyping, Normal DHR | WES |  |  | No pathogenic variant |  |
| 177 | No | 188 | M | MSMD | Normal immunophenotyping, Normal DHR | WES |  |  | No pathogenic variant |  |
| 178 | Yes | 189 | F | MSMD | Normal immunophenotyping, Normal DHR | WES |  |  | No pathogenic variant |  |
| 179 | Yes | 190 | F | MSMD | Normal immunophenotyping, Normal DHR | WES |  |  | No pathogenic variant |  |
| 180 | Yes | 191 | F | MSMD | Normal immunophenotyping, Normal DHR | WES |  |  | No pathogenic variant |  |
| 181 | No | 192 | F | MSMD | Normal immunophenotyping, Normal DHR | WES |  |  | No pathogenic variant |  |
| 182 | No | 193 | F | MSMD | Normal immunophenotyping, Normal DHR | WES |  |  | No pathogenic variant |  |
| 183 | No | 194 | F | MSMD | Normal DHR | WES |  |  | No pathogenic variant |  |
| 184 | No | 195 | M | MSMD | Normal immunophenotyping, Normal DHR | WES |  |  | No pathogenic variant |  |
| 185 | Yes | 196 | F | MSMD | Normal immunophenotyping | WES |  |  | No pathogenic variant |  |
| 186 | Yes | 197 | F | ALPS-like | Normal immunophenotyping, Normal DHR | WES |  |  | No pathogenic variant |  |
| 187 | No | 198 | M | Recurrent pyogenic arthritis/CGD? | Normal immunophenotyping, Normal DHR | WES |  |  | No pathogenic variant |  |
| 188 | Yes | 199 | F | CGD | Defective DHR, Defective NCF1 | Sanger | *NCF1* | AR | Exon 2,9 No pathogenic variant |  |
| 189 | No | 200 | M | CGD | Defective DHR, Defective NCF1 | Sanger | *NCF1* | AR | Exon 2,9 No pathogenic variant |  |
| 190 | Yes | 201 | M | CGD | Defective DHR | Sanger | *NCF1,NCF2, CYBA* | AR | No pathogenic variant |  |
|  |  |  |  |  |  |  | *CYBB* | XL | No pathogenic variant |  |
| 191 | Yes | 202^a^ | F | CGD | Defective DHR | Sanger | *NCF1,NCF2,CYBA* | AR | No pathogenic variant |  |
|  |  | 203^b^ | F | CGD | Defective DHR | Sanger | *NCF1,NCF2,CYBA* | AR | No pathogenic variant |  |
| 192 | Yes | 204 | M | CGD | Defective DHR,  bimodal maternal DHR pattern | Sanger | *CYBB* | XL | Pending |  |
| 193 | Yes | 205 | M | CGD | Defective DHR, intracellular NCF1 | Sanger | *NCF1* | AR | Exon 2,9 No pathogenic variant |  |
| 194 | Yes | 206 | M | CGD | Defective DHR, intracellular NCF1 | Sanger | *NCF1* | AR | Exon 2,9 No pathogenic variant |  |
| 195 | No | 207 | F | CGD-like | Defective DHR, normal CYBA, NCF1,NCF4 | WES |  |  | No pathogenic variant |  |
| 196 | No | 208 | M | CGD-like | Normal immunophenotyping, Normal DHR | WES |  |  | No pathogenic variant |  |
| 197 | No | 209 | M | Lymphoproliferative disorder | Increase αβ double negative T cells | NGS |  |  | No pathogenic variant |  |
| 198 | No | 210 | F | Lymphoproliferative disorder | Increase αβ double negative T cells | NGS |  |  | No pathogenic variant |  |
| 199 | Yes | 211 | M | Lymphoproliferative disorder | Increase αβ double negative T cells | NGS |  |  | No pathogenic variant |  |
| 200 | No | 212 | M | Lymphoproliferative disorder | Increase αβ double negative T cells | NGS |  |  | No pathogenic variant |  |
| 201 | No | 213 | F | Neutropenia | Low CD19, Absolute neutropenia | WES |  |  | No pathogenic variant |  |
| 202 | No | 214 | F | Neutropenia | Normal immunophenotype, Absolute neutropenia | WES |  |  | Pending |  |
| 203 | No | 215 | M | Neutropenia | Normal immunophenotype, Absolute neutropenia | WES |  |  | Pending |  |
| 204 | No | 216 | F | Neutropenia | Normal immunophenotype, Absolute neutropenia | WES |  |  | Pending |  |
| 205 | No | 217 | M | Neutropenia | Normal CD 55 & CD 59 | WES |  |  | Pending |  |
| 206 | No | 218 | M | WHIM | Low CD3 , Neutropenia | Sanger | *CXCR4* | AR | No pathogenic variant |  |
| 207 | Yes | 219 | F | Bone marrow failure syndrome,  Necrotizing skin granulomas | Absolute lymphopenia, Normal DHR  Low CD3,CD4,CD8,CD19,CD56 | FISH | Chr. 7 |  | Normal |  |
|  |  |  |  |  |  | FISH | Chr. q5 |  | Normal |  |
| 208 | Yes | 220 | M | Bone marrow failure syndrome | BM biopsy | NGS |  |  | No pathogenic variant |  |
| 209 | Yes | 221 | F | HLH | BM asprite | WES |  |  | No pathogenic variant |  |
| 210 | Yes | 222 | M | Autoinflammatory | Normal immunophenotype | NGS |  |  | No pathogenic variant |  |
